# N-glycosylation by Mgat5 imposes a targetable constraint on immune-mediated tumor clearance

**DOI:** 10.1172/jci.insight.178804

**Published:** 2024-05-23

**Authors:** Erin E. Hollander, Rosemary E. Flock, Jayne C. McDevitt, William P. Vostrejs, Sydney L. Campbell, Margo I. Orlen, Samantha B. Kemp, Benjamin M. Kahn, Kathryn E. Wellen, Il-Kyu Kim, Ben Z. Stanger

**Affiliations:** 1 Department of Medicine and; 2 Abramson Cancer Center and Abramson Family Cancer Research Institute, Perelman School of Medicine, University of Pennsylvania, Philadelphia, Pennsylvania, USA.; 3 Department of Biology and; 4 Department of Cancer Biology, University of Pennsylvania, Philadelphia, Pennsylvania, USA.

**Keywords:** Cell biology, Oncology, Apoptosis, Glycobiology, T cells

## Abstract

The regulated glycosylation of the proteome has widespread effects on biological processes that cancer cells can exploit. Expression of N-acetylglucosaminyltransferase V (encoded by *Mgat5* or *GnT-V*), which catalyzes the addition of β1,6-linked N-acetylglucosamine to form complex N-glycans, has been linked to tumor growth and metastasis across tumor types. Using a panel of murine pancreatic ductal adenocarcinoma (PDAC) clonal cell lines that recapitulate the immune heterogeneity of PDAC, we found that *Mgat5* is required for tumor growth in vivo but not in vitro. Loss of *Mgat5* results in tumor clearance that is dependent on T cells and dendritic cells, with NK cells playing an early role. Analysis of extrinsic cell death pathways revealed *Mgat5*-deficient cells have increased sensitivity to cell death mediated by the TNF superfamily, a property that was shared with other non-PDAC *Mgat5*-deficient cell lines. Finally, *Mgat5* knockout in an immunotherapy-resistant PDAC line significantly decreased tumor growth and increased survival upon immune checkpoint blockade. These findings demonstrate a role for N-glycosylation in regulating the sensitivity of cancer cells to T cell killing through classical cell death pathways.

## Abstract 2

The *N*-glycosyltransferase Mgat5 inhibits the ability of a T cell to kill its target, rendering immunotherapy less effective in a mouse model of pancreatic cancer.

## Graphical abstract

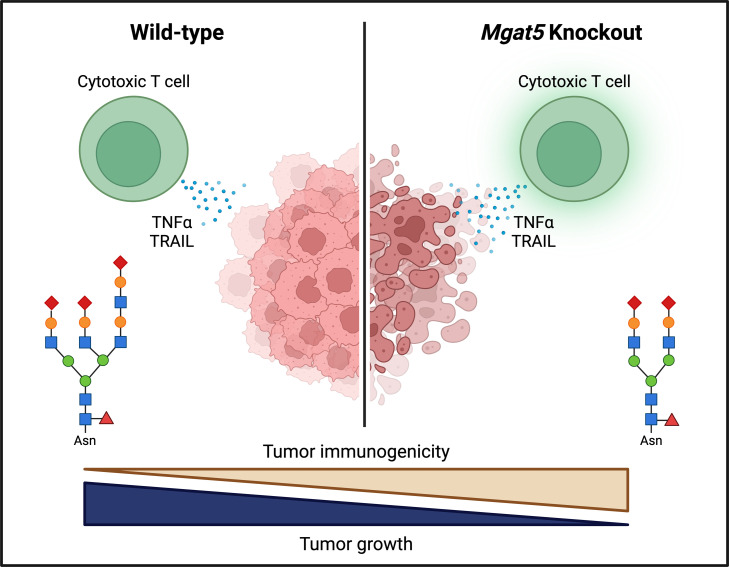

## Introduction

Glycosylation — the regulated decoration of proteins and lipids with various sugar moieties — influences almost every aspect of cell biology (1, 2). Altered glycosylation is a hallmark of cancer, as tumor cells dysregulate glycosylation pathways as a means of altering cell adhesion and motility (3), epithelial-to-mesenchymal state (4), immune receptor activation and inhibition (5, 6), sensitivity to immune checkpoint blockade (ICB) (7), and other cellular processes (8). As glycosylation involves a stepwise series of enzymatic steps in the endoplasmic reticulum and Golgi, its frequent dysregulation in cancer may represent a targetable vulnerability (9, 10).

The glycosyltransferase Mgat5 (α-1,6-mannosylglycoprotein 6-β-*N*-acetylglucosaminyltransferase V) is responsible for terminal branching of complex N-glycans, which it achieves through the addition of N-acetylglucosamine (GlcNAc) from UDP-GlcNAc in a β-1,6 linkage to a core mannose (11, 12). Overexpression of *Mgat5* has been linked to tumor aggressiveness and poor prognosis in multiple cancer types, including colorectal, endometrial, hepatocellular, and gastric cancers (13–16), whereas loss of *Mgat5* has been associated with decreased tumor growth in multiple cancers both in vitro and in vivo, including colorectal cancer (13, 17), breast cancer (18, 19), nasopharyngeal carcinoma (20), lung adenocarcinoma (21), hepatocellular carcinoma (HCC) (22–24), and gastric cancer (25). The mechanisms by which Mgat5 alters tumor growth and interactions with the immune system are not fully understood.

Pancreatic ductal adenocarcinoma (PDAC) has a 5-year survival rate of only 13% (26). This poor outcome is due to the advanced nature of most tumors at presentation and limited options for treating systemic disease (27–29). In particular, PDAC tumors commonly possess an immunosuppressive tumor microenvironment (TME) (30, 31) and are resistant to ICB (32, 33). PDAC tumors also exhibit global changes in glycosylation, including a well-recognized increase in the abundance of the sialyl Lewis^A^ antigen CA19-9 (34); in addition, PDACs have increased fucosylated and branched N-glycans (35, 36) and truncated O-glycans (37), both of which are associated with tumor progression and poor prognosis. The role of *Mgat5* in PDAC tumor biology has not been explored.

Using PDAC as a model, we examined the consequences of eliminating *Mgat5* from pancreatic cancer cells. Whereas *Mgat5*-deficient cells exhibited normal growth in vitro and in immunodeficient hosts in vivo, tumor formation was almost completely abolished in immunocompetent hosts in vivo. Clearance of *Mgat5*-deficient cells was mediated by T cells and could be attributed in part to an increase in sensitivity to cell apoptosis and necroptosis. These findings highlight the importance of Mgat5-mediated glycosylation in the T cell response to tumor cells and support the development of Mgat5 inhibitors as potential immune-sensitizing agents.

## Results

### Mgat5 loss sensitizes cancer cells to immune clearance.

**Figure 1:**
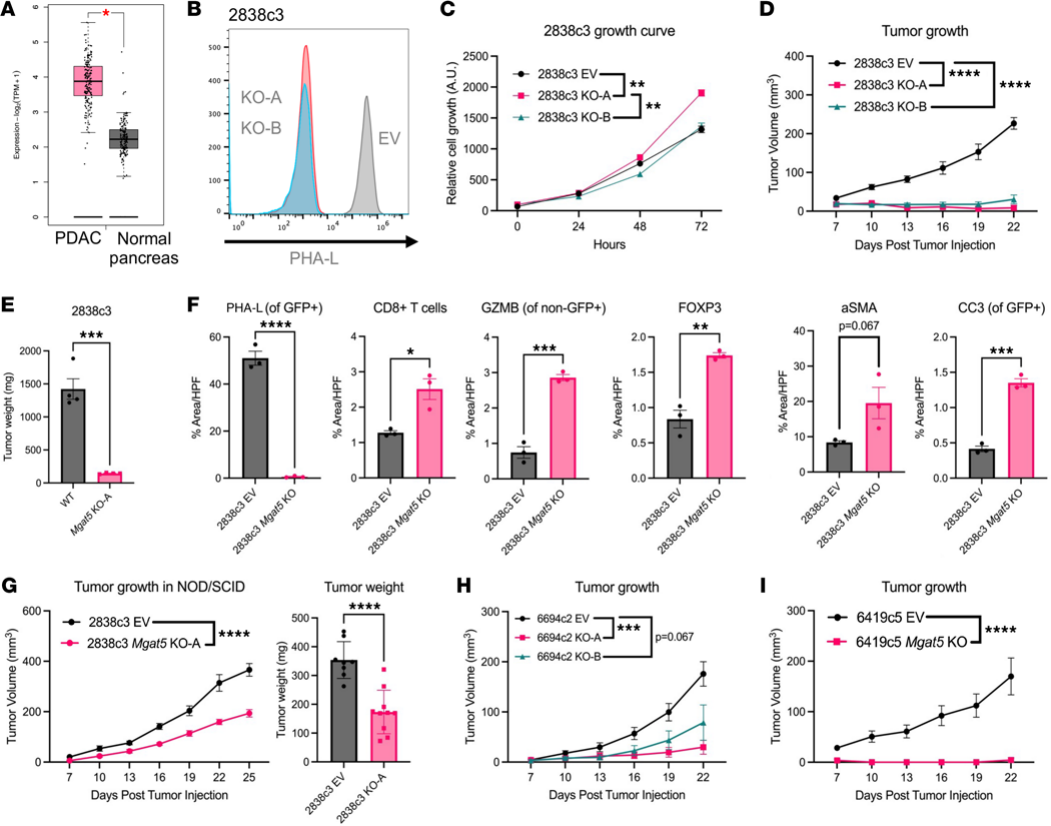
*Mgat5* loss sensitizes cancer cells to immune clearance. (**A**) *Mgat5* expression in human pancreatic cancer (left, *n* = 179) and normal pancreas (right, *n* = 171) from GEPIA2. In the box-and-whisker plot, the lower and upper bounds of the box represent the 25th and 75th percentiles of the data, respectively, with the line within the box set at the median. Whiskers are set at the lowest and greatest values in the data set, excluding the outliers plotted beyond the whiskers. (**B**) Histogram of PHA-L binding to 2838c3 EV and *Mgat5*-KO cell lines. (**C**) Relative growth of T cell–inflamed (2838c3) EV and *Mgat5*-KO cell lines in vitro. Statistical analysis done using 2-way ANOVA with *n* = 3 replicates per data point. (**D**) Growth (mm^3^) of 2838c3 EV (*n* = 6), *Mgat5*-KO-A (*n* = 7), and *Mgat5*-KO-B (*n* = 7) subcutaneous tumors in *C57BL/6* mice over time. Statistical analysis done using 2-way ANOVA for this and all following tumor growth curves. Data representative of 2 independent experiments. (**E**) Tumor weights in T cell–inflamed (2838c3) WT (*n* = 4) and *Mgat5*-KO-A (*n* = 4) cells implanted orthotopically into mouse pancreas. Data represent mean ± SEM. Statistical analysis using unpaired, 2-tailed Student’s *t* test. (**F**) Quantification of immunofluorescent staining for PHA-L, CD8^+^ T cells, granzyme B (GZMB), FOXP3, aSMA, and cleaved caspase 3 (CC3) by percentage area per high-power field (HPF) using ImageJ. Three mice per condition (EV, KO) with 3–5 HPF per tumor. Statistics using unpaired, 2-tailed Student’s *t* test. (**G**) Growth (mm^3^) and weights (mg) of 2838c3 EV (*n* = 8) and *Mgat5*-KO-A (*n* = 10) subcutaneous tumors in NOD/SCID mice. Data represent mean ± SEM. (**H**) Growth (mm^3^, left) of 6694c2 EV (*n* = 8), *Mgat5*-KO-A (*n* = 10), and *Mgat5*-KO-B (*n* = 10) subcutaneous tumors in *C57BL/6* mice. Data represent mean ± SEM. (**I**) Subcutaneous tumor growth over time of 6419c5 EV (*n* = 6) and *Mgat5*-KO (*n* = 6) tumors in *C57BL/6* mice. Data representative of 2 independent experiments. Data represent mean ± SEM. *P < 0.05; **P < 0.01; ***P < 0.001; ****P < 0.0001.

Analysis of The Cancer Genome Atlas Program (TCGA) and Genotype-Tissue Expression (GTex) project revealed significantly increased *Mgat5* expression in human PDAC samples as compared with normal pancreas samples (Figure 1A). To study the function of *Mgat5* in an immunocompetent setting, the *KPC/Y* mouse model of PDAC (KRAS^G12D^, p53^R172H^, Cre^Pdx^, YFP) was utilized, which gives rise to heterogeneous tumors with varying histologies and degrees of immune infiltration (38, 39). We previously reported on a panel of clonal *KPC/Y* cell lines on the *C57BL/6* background that recapitulate the immune heterogeneity in PDAC. When implanted into syngeneic immunocompetent hosts, T cell–inflamed tumor cell clones give rise to tumors characterized by an immunogenic TME containing CD4^+^ and CD8^+^ T cells and a paucity of granulocytic myeloid–derived suppressor cells (gMDSCs), while non–T cell–inflamed clones give rise to tumors characterized by a paucity of T cells and an abundance of gMDSCs (40).

To examine the role of *Mgat5* in PDAC tumor cell biology, we used CRISPR to inactivate *Mgat5* in tumor cells from the T cell–inflamed clonal line 2838c3. Successful knockout (KO) was confirmed by flow cytometry using *Phaseolus* *vulgaris* lectin L (PHA-L), which probes for Mgat5-mediated N-glycans (Figure 1B). *Mgat5*-deficient lines (KO-A and KO-B) derived from the 2838c3 T cell–inflamed clone had no growth deficiency compared to empty vector (EV) controls in vitro (Figure 1C). In contrast, subcutaneous injection of 2838c3 *Mgat5*-KO cells into *C57BL/6* hosts resulted in an initial period of growth followed by the near-complete elimination of tumors 2 weeks after injection. After 3 weeks, *Mgat5*-deficient tumors were significantly smaller than EV controls (Figure 1D and Supplemental Figure 1A; supplemental material available online with this article; https://doi.org/10.1172/jci.insight.178804DS1). Clearance of *Mgat5*-KO tumors was validated following orthotopic implantation into the pancreas (Figure 1E).

Next, we analyzed the tissues histologically to assess changes in tumor architecture. Hematoxylin & eosin (H&E) staining of T cell–inflamed EV and *Mgat5*-KO tumors harvested 12 days after injection (the time of maximal tumor size) revealed no obvious differences between KO and EV tumors (Supplemental Figure 1B). Immunofluorescence (IF) for PHA-L confirmed *Mgat5* KO in vivo (Figure 1F and Supplemental Figure 1B), while further staining revealed significant increases in tumor-infiltrating CD8^+^ T cells (Figure 1F and Supplemental Figure 1B). A significant increase in non–tumor cell granzyme B content was observed, suggesting an increase in T cell and NK cell cytotoxicity against tumor cells (Figure 1F). Regulatory T cells (Tregs), as captured by FOXP3 expression, were increased in *Mgat5*-KO tumors (Figure 1F). *Mgat5* KO in the T cell–inflamed line resulted in a nonsignificant trend toward increased α-smooth muscle actin^+^ (aSMA^+^) fibroblasts (Figure 1F). In addition, the apoptotic marker cleaved caspase-3 (CC3) was significantly increased in *Mgat5*-KO tumor cells, indicating increased cancer cell death (Figure 1F and Supplemental Figure 1B).

To functionally examine the role of immune pressure on the growth of *Mgat5*-deficient tumors, we subcutaneously implanted EV or *Mgat5*-KO tumor cells from a T cell–inflamed clone (2838c3) into *NOD/SCID* mice lacking functional T cells, B cells, and NK cells. In contrast with the growth inhibition phenotype observed in immunocompetent *C57BL/6* mice, *Mgat5*-KO tumors were able to grow in *NOD/SCID* mice, albeit not to the same degree as EV control cells (Figure 1G). *Mgat5*-KO cells were no more sensitive than EV controls to growth in low glucose or low serum — conditions reflecting the more nutrient-deprived TME in vivo (Supplemental Figure 2, A and B). Collectively, these results suggest that immune-mediated clearance is responsible for the growth deficiency associated with *Mgat5* loss, with nonimmune factors also playing a role.

### Mgat5 loss increases immune clearance in immunosuppressive microenvironments.

Human pancreatic tumors typically harbor immunosuppressive microenvironments that hinder the efficacy of immunotherapies such as CAR T cells and ICB (31, 32, 41, 42). Overcoming this immunologic barrier is paramount to expanding options for treatment. To determine the effects of *Mgat5* loss on tumor clearance in an immunosuppressive microenvironment, we used CRISPR to KO *Mgat5* in 2 non–T cell–inflamed (“cold”) clonal cell lines (6694c2 and 6419c5), which reflect the myeloid-rich TME of most PDAC tumors. Although our clonal PDAC lines exhibit different levels of *Mgat5* RNA expression at baseline, all produce similar overall levels of Mgat5-derived N-glycans as assessed by PHA-L binding (Supplemental Figure 3, A and B). Following CRISPR-mediated gene editing, loss of *Mgat5* was again confirmed using PHA-L staining (Supplemental Figure 3C). Similar to our observations in the T cell–inflamed line 2838c3, the growth of non–T cell–inflamed lines 6694c2 and 6419c5 lacking *Mgat5* was comparable to that of EV controls in vitro (Supplemental Figure 3D, but significantly reduced in vivo (Figure 1, H and I, and Supplemental Figure 3E). Specifically, no tumors formed following implantation of the *Mgat5*-KO 6419c5 non–T cell–inflamed line, whereas tumors grew only half of the time following implantation of the *Mgat5*-KO 6694c2 non–T cell–inflamed lines (Table 1). Thus, loss of *Mgat5* results in a profound inability of PDAC tumor cells to grow in vivo in the setting of both T cell–inflamed and non–T cell–inflamed tumors.

We then performed a histological analysis of non–T cell–inflamed EV and *Mgat5*-KO tumors 12 days after implantation. As with our T cell–inflamed tumors (Supplemental Figure 1B), H&E staining failed to show significant histological differences between *Mgat5*-KO and EV control tumors (Supplemental Figure 4A). IF for PHA-L confirmed loss of Mgat5 activity in KO tumors (Supplemental Figure 4, A and B). CD8^+^ T cells and Treg cells (FOXP3^+^) were increased in *Mgat5*-KO tumors, as was granzyme B expression (Supplemental Figure 4, A and B). Staining for aSMA revealed a significant increase in myofibroblasts, while staining for CC3 in tumor cells revealed a significant increase in apoptotic cell death (Supplemental Figure 4, A and B). These results suggest that *Mgat5* loss in immunosuppressive, non–T cell–inflamed tumors is accompanied by an increase in tumor-infiltrating T cells, myofibroblasts, and tumor cell death.

### Mgat5 glycans allow cancer cells to evade T cell–mediated clearance.

**Figure 2:**
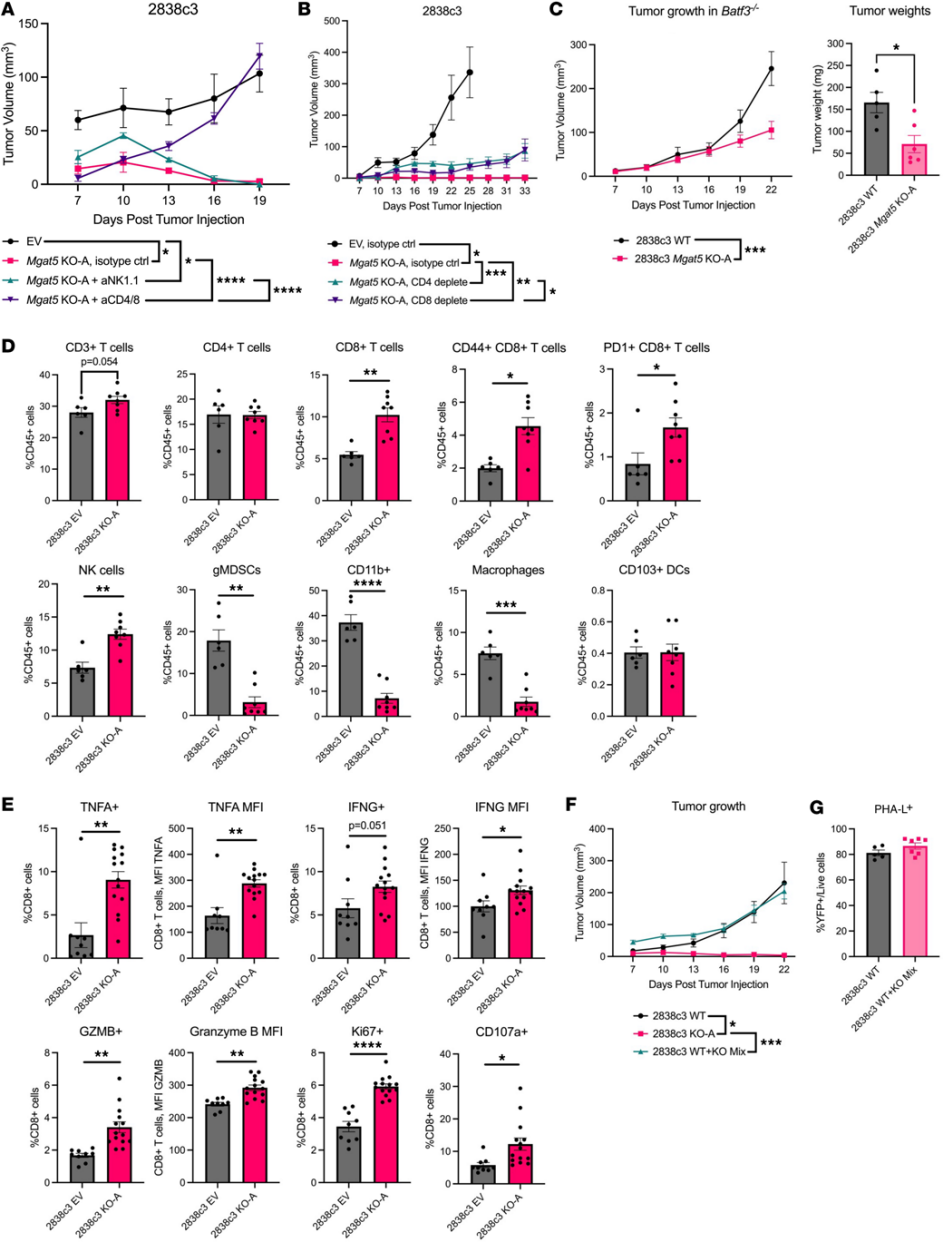
Mgat5 glycans help cancer cells evade clearance by T cells in a cell-autonomous manner. (**A**) Tumor volumes (mm^3^) over time of 2838c3 EV (*n* = 5) and *Mgat5*-KO-A subcutaneous tumors in the setting of either NK cell depletion (*n* = 10), CD4^+^/CD8^+^ T cell depletion (*n* = 10), or isotype control (*n* = 5). Data represent mean ± SEM. Statistical analysis done using 2-way ANOVA for this and all further tumor growth curves. (**B**) Growth (mm^3^) of 2838c3 EV and *Mgat5*-KO-A subcutaneous tumors in the setting of either CD4^+^ T cell depletion, CD8^+^ T cell depletion, or isotype control (*n* = 5 mice/group). Statistical analysis done on day 19. (**C**) Growth (mm^3^, left) and weights (right) of 2838c3 EV (*n* = 5) and *Mgat5*-KO-A (*n* = 6) subcutaneous tumors in *Batf3*^–/–^ mice. Statistical analysis done using unpaired, 2-tailed Student *t* test for tumor weights. Data representative of 2 independent experiments. (**D**) Flow cytometric analysis of the indicated immune cell subsets in 2838c3 EV (*n* = 6) and *Mgat5*-KO-A tumors (*n* = 8) harvested on day 12 after subcutaneous injection. Data represent mean ± SEM. Statistical analysis using unpaired, 2-tailed Student’s *t* test. Data representative of 2 independent experiments. (**E**) Flow cytometric analysis of T cell cytotoxicity in draining lymph nodes (inguinal, axillary) from 2838c3 EV and *Mgat5*-KO subcutaneous flank tumor. Data represent mean ± SEM. Statistical analysis using unpaired, 2-tailed Student’s *t* test. Data representative of 2 independent experiments. (**F**) Growth (mm^3^) tumors following the injection of 2838c3 WT cells (*n* = 6), *Mgat5*-KO-A cells (*n* = 4), or a 1:1 mix of WT and KO cells (*n* = 7). (**G**) PHA-L staining by flow cytometry of the WT and 1:1 mixed WT + KO tumors. Statistical analysis done using unpaired, 2-tailed Student’s *t* test. *P < 0.05; **P < 0.01; ***P < 0.001; ****P < 0.0001.

*NOD/SCID* mice contain an array of immunodeficiencies that could have contributed to the rescue of *Mgat5*-KO tumor growth. To explore which immune populations mediate tumor rejection, we injected immunodepleting antibodies against either CD4^+^ and CD8^+^ T cells or NK cells into *C57BL/6* mice bearing either T cell–inflamed EV or *Mgat5*-KO tumor cells (2838c3). Flow of cytometric analysis of cells from spleen (CD4^+^ and CD8^+^ T cells) or liver (NK cells) confirmed efficient depletion (Supplemental Figure 5, A–C). CD4^+^/CD8^+^ T cell depletion led to full rescue of *Mgat5*-KO tumor growth (Figure 2A). By contrast, NK cell depletion had no effect on the ultimate rejection of the KO tumors, although it did result in a small and reproducible enhancement of tumor growth on day 10 after injection (Supplemental Figure 5D). To further explore the role of T cells in the rejection of *Mgat5*-deficient tumors, we depleted CD4^+^ and CD8^+^ T cells separately and found that each depletion led to a partial rescue of *Mgat5*-KO tumor growth (Figure 2B). These data suggest that both CD4^+^ and CD8^+^ T cells are important for the enhanced clearance of *Mgat5*-deficient tumor cells.

Next, we asked whether dendritic cells (DCs) or B cells contribute to the *Mgat5*-KO growth phenotype. To this end, we subcutaneously implanted *Mgat5*-KO and control wild-type (WT) tumor cells into *Batf3*^–/–^ hosts, which lack conventional type 1 DCs (cDC1s) that are specialized for CD8^+^ T cell activation and cross-presentation (43). Tumor implantation into *Batf3*^–/–^ mice rescued the *Mgat5*-KO growth deficiency that was most pronounced at early time points (Figure 2C). By contrast, *muMT*^–^ host mice lacking B cells fully cleared *Mgat5*-KO tumors (Supplemental Figure 5E). Taken together, these results suggest that CD4^+^ and CD8^+^ T cells, and cDC1s, play a dominant role in the clearance of *Mgat5*-deficient tumors.

### Mgat5 deficiency confers a tumor cell–autonomous sensitivity to immune pressure.

To gain further insights into immune changes within the TME, we performed immune profiling of T cell–inflamed (2838c3) EV and *Mgat5*-KO tumors (Supplemental Figure 6A). Given that our standard injection approach resulted in *Mgat5*-KO tumors that were too small for immune profiling (even at the point of maximal size; 12 days), 10-fold more tumor cells (2 × 10^6^) were injected into *C57BL/6* mice to obtain tumors that would be adequate for flow cytometry. *Mgat5*-deficient tumors exhibited a significant increase in total T cells (CD3^+^), CD8^+^ T cells, and NK cells; the activation marker CD44 and exhaustion marker PD-1 were both significantly upregulated on CD8^+^ T cells (Figure 2D). In addition, *Mgat5*-deficient tumors exhibited a significant decrease in myeloid cells (CD45^+^CD11b^+^), macrophages (CD11b^+^F4/80^+^), and gMDSCs (Ly-6G^+^Ly-6C^+^). Similar changes were observed in *Mgat5*-KO tumors derived from a non–T cell–inflamed line (6694c2) (Supplemental Figure 7A).

To further deconstruct the immune response to *Mgat5*-KO tumors, draining lymph nodes (inguinal, axillary) from subcutaneous flank tumors were isolated 12 days after tumor cell injection. This allows for dissection of the T cell response at a more granular level than is possible in primary tumors, given limited tumor-infiltrating lymphocytes. Markers of T cell cytotoxicity, including TNF-α, IFN-γ, and granzyme B were significantly upregulated in both CD8^+^ T cells (Figure 2E) and CD4^+^ T cells (Supplemental Figure 7B) within the draining lymph node, along with significant increases in T cell (Ki67^+^) and degranulation (CD107a/LAMP-1). These results indicate that the T cells that infiltrate *Mgat5*-KO tumors are activated and functional in the immune response.

We next determined whether the eradication of *Mgat5*-KO tumors can be attributed to the changes in the TME or cell-autonomous effects induced by *Mgat5* deficiency. We reasoned that if changes in the TME brought about by *Mgat5* deficiency underlie the phenotype, then such changes in immune infiltration should also result in the eradication of cells with intact *Mgat5*. To this end, we mixed WT and *Mgat5*-KO cells at a 1:1 ratio and subcutaneously injected them into *C57BL/6* mice. The resulting tumors from the mixed injection grew at a comparable rate as the WT controls (Figure 2F). However, most surviving tumor cells were PHA-L^+^ (Figure 2G), indicative of an immune response that cleared the *Mgat5*-KO cells and spared the *Mgat5*-WT cells. These results suggest that the immune-mediated elimination of *Mgat5*-deficient cells is cell autonomous and that the observed changes in immune infiltration are a consequence rather than a cause of the tumor rejection mechanism.

### Loss of Mgat5 increases the immunogenicity of existing tumor antigens.

We considered several possible mechanisms by which loss of *Mgat5* could lead to improved T cell recognition and killing. These included (i) the creation of neoantigens, potentially via the addition of new sugar moieties to (normally) unmodified mannose moieties; (ii) uncloaking of existing antigens whose processing and presentation are blocked via steric hindrance in *Mgat5*-WT cells; and (iii) changes in tumor cell glycosylation resulting an enhanced ability of T cells to recognize and/or kill target cells.

**Figure 3:**
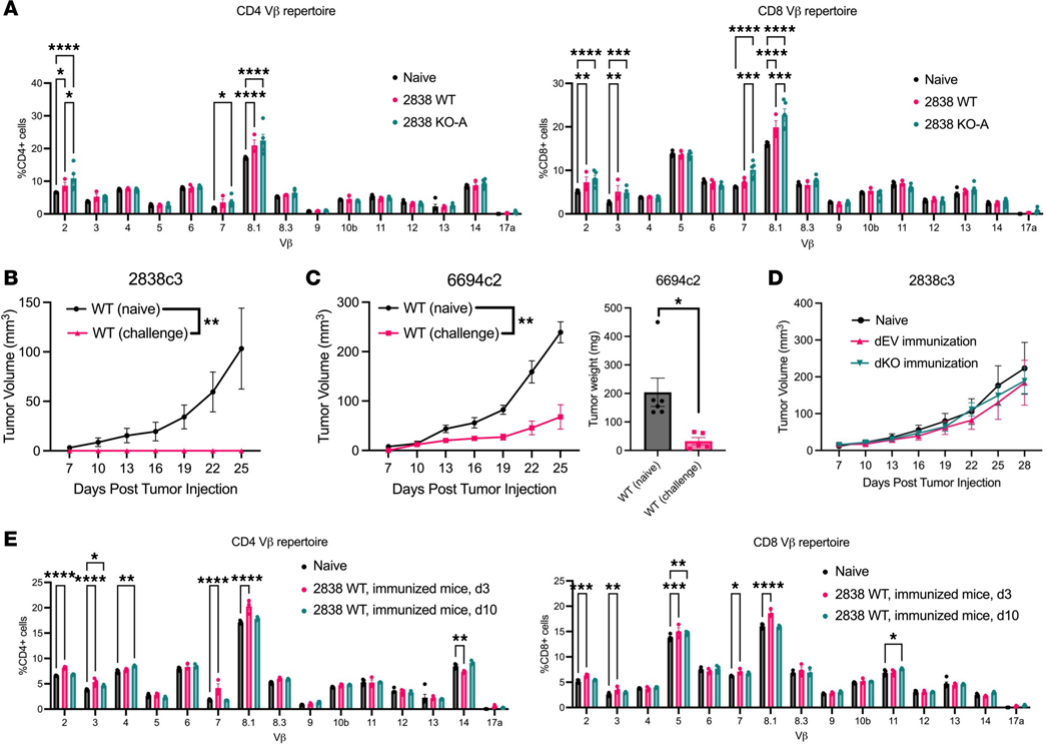
*Mgat5* loss increases the immunogenicity of existing tumor antigens. (**A**) Vβ repertoire analysis of CD4^+^ and CD8^+^ T cells in the spleens of naive mice (*n* = 5, combined from 2 independent experiments), mice bearing 2838c3 WT tumors (*n* = 3), or mice that had cleared *Mgat5*-KO-A tumors (*n* = 5). Data represent mean ± SEM. Statistical analysis by 2-way ANOVA for this and all further tumor growth curves. (**B**) Growth (mm^3^) of subcutaneous tumors arising from 2838c3 WT cells injected into either naive mice or mice previously immunized (4 weeks earlier) with 2838c3 *Mgat5*-KO-A cells (*n* = 6 mice/group). Data represent mean ± SEM. (**C**) Growth (mm^3^, left) and weights (right) of 6694c2 WT cells subcutaneous injected into either naive mice or mice previously immunized (4 weeks earlier) with 2838c3 *Mgat5*-KO-A cells (*n* = 6 mice/group). Data represent mean ± SEM. Statistical analysis by unpaired, 2-tailed Student *t* test for tumor weights. (**D**) Growth (mm^3^) of 2838c3 WT cells subcutaneously injected into naive mice, mice previously immunized with irradiated, dead 2838c3 EV cells (dEV immunization), or mice previously immunized with dead 2838c3 KO-A cells (dKO immunization) (*n* = 7 mice/group). Data represent mean ± SEM. (**E**) Vβ repertoire analysis of CD4^+^ and CD8^+^ T cells in the spleens of naive mice (*n* = 5, from **A**), immunized mice challenged with 2838c3 WT tumor cells day 3 after subcutaneous injection (*n* = 3), or immunized mice challenged with 2838c3 WT tumor cells on day 10 after subcutaneous injection (*n* = 3). Data represent mean ± SEM. Statistical analysis by 2-way ANOVA. *P < 0.05; **P < 0.01; ***P < 0.001; ****P < 0.0001.

To address the possibility that the immune system was responding to neoantigens emerging in *Mgat5*-KO cells (possibilities i and ii), we examined T cell repertoires for evidence of T cell subclones specific for *Mgat5*-KO tumors. To this end, we isolated splenocytes from naive *C57BL/6* mice or mice injected with *Mgat5*-WT or *Mgat5*-KO tumor cells for T cell receptor Vβ repertoire analysis of both CD4^+^ and CD8^+^ T cells. A review of the T cell repertoires revealed an enrichment of several Vβ receptors in tumor-bearing mice compared with naive *C57BL/6* controls, notably Vβ2, Vβ3, Vβ7, and Vβ8.1 (Figure 3A). Notably, these same Vβ receptor subtypes were enriched in both CD4^+^ and CD8^+^ T cells, suggesting a polyclonal T cell response to tumor-associated antigens in *Mgat5*-WT tumors.

Next, we examined mice bearing *Mgat5*-KO tumors to determine whether there was an expansion of T cells expressing Vβ alleles that differed from naive and WT tumor–bearing mice, which might reflect a T cell response to antigens present exclusively in *Mgat5*-KO cells. Our analysis failed to reveal changes in the Vβ repertoire fitting this pattern. Instead, the T cell subsets enriched in response to *Mgat5*-KO tumors were the same as those that had expanded in response to *Mgat5*-WT tumors (Figure 3A). These results suggest that *Mgat5*-deficient cancer cells do not provoke a T cell response against a new set of antigens, but rather increase the strength of the response to preexisting antigens present in *Mgat5*-WT cells.

Given these findings, we hypothesized that *Mgat5* deficiency results in improved immunogenicity, possibly by enhancing T cell–target cell interactions. To test this hypothesis, we performed tumor immunization studies to determine whether live cell interactions are required for the immune response to *Mgat5*-KO cells. First, *C57BL/6* mice were subcutaneously injected with 2838c3 *Mgat5*-KO cells, and tumors were allowed to fully clear over 4 weeks. These “cured” mice were then challenged with WT cells, using naive mice injected with *Mgat5*-WT cells as a control. Mice previously injected (immunized) with *Mgat5*-KO cells were able to fully clear parental T cell–inflamed *Mgat5*-WT cells (Figure 3B) and to control the growth of an unrelated non–T cell–inflamed *Mgat5*-WT line (6694c2) (Figure 3C), indicating that the strong immune response to the *Mgat5*-deficient cells generated memory against antigens present across the PDAC clonal lines, as previously described (40). We then used 2 methods (sonication and ionizing radiation) to kill EV and *Mgat5*-KO cells. Mice were then immunized with the dead cells and then challenged with WT cells using identical methodology as described above. In contrast with the protective effects conferred by live *Mgat5*-KO cells, mice immunized with dead EV or *Mgat5*-KO cells were susceptible to tumor rechallenge, with *Mgat5*-WT cells able to grow at the same rate as they did in a naive host (Figure 3D and Supplemental Figure 8A). These findings indicate that a live tumor cell–T cell interaction is necessary for the strong immune reaction against *Mgat5*-KO cells; likewise, these data fail to provide evidence that a strong tumor antigen is created upon *Mgat5* loss.

Returning to Vβ repertoire analysis, we immunized mice with 2838c3 *Mgat5*-KO cells as above and then challenged them with 2838c3 WT cells. As noted in Figure 3B, clearance of the WT tumors in immunized mice occurs prior to the initiation of tumor measurements at 7 days after injection. We therefore evaluated the CD4^+^ and CD8^+^ T cell receptor repertoire at 3 and 10 days after injection. Notably, the same receptor subtypes (Vβ2, Vβ7, and Vβ8.1) that were expanded upon clearance of *Mgat5*-KO tumors (Figure 3A) were also found to be expanded on day 3 after challenge with *Mgat5*-WT tumor cells, along with Vβ3 and Vβ5 (Figure 3E). These expansions returned to the naive baseline by day 10, suggesting that these T cell subsets are responsible for responding to and clearing the tumors. These results lend further support to the notion that *Mgat5* loss does not lead to the creation of a strong tumor antigen that evokes a T cell response; rather, an enhancement of the immunogenicity of existing tumor antigens appears to allow for a polyclonal T cell response and robust immune memory.

### Mgat5 deficiency results in enhanced sensitivity to TNF-α– and TRAIL-mediated cell death.

**Figure 4:**
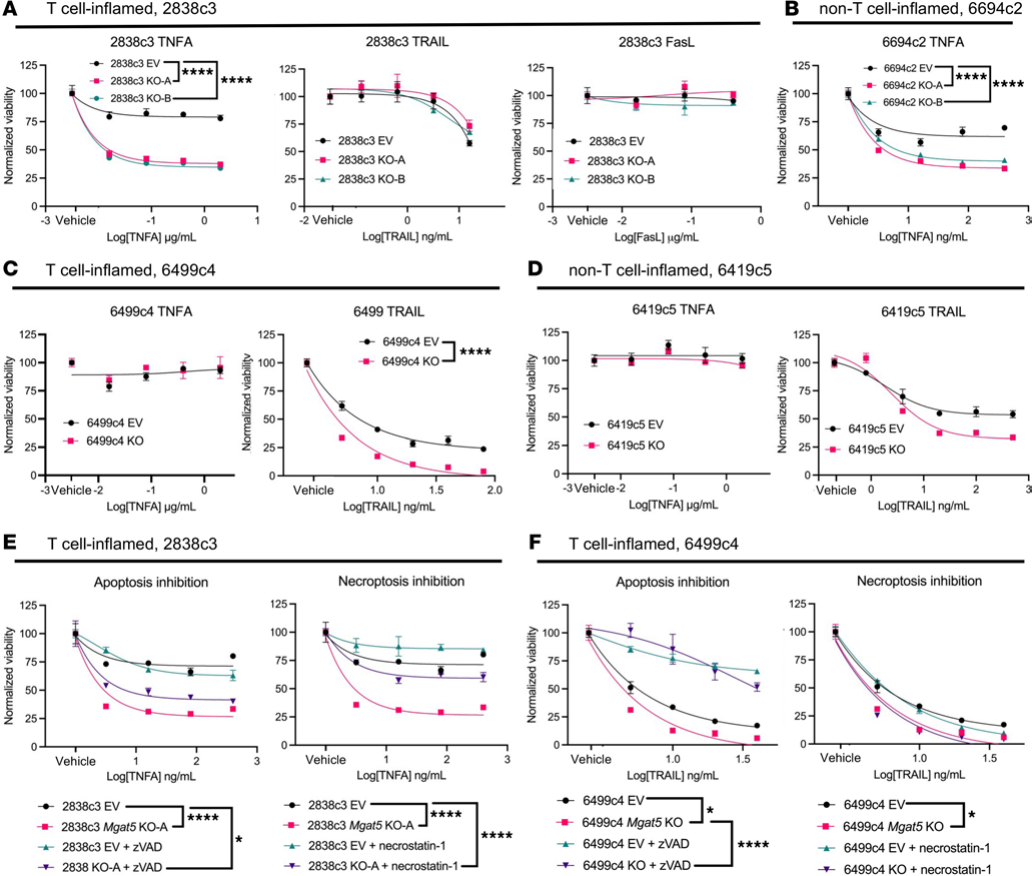
Mgat5 glycans impede cell killing via extrinsic cell death pathways. (**A**) Viability of 2838c3 EV and *Mgat5*-KO cells in vitro following exposure to varying concentrations of TNF-α, TRAIL, or Fas ligand (FasL). Statistics from 2 μg/mL TNF-α, 16 ng/mL TRAIL, and 0.4 μg/mL FasL. Analysis using 2-way ANOVA for this and all further cell death figures at specified concentrations. (**B**) Viability of 6694c2 EV and *Mgat5*-KO cells in vitro following exposure to varying concentrations of TNF-α. Statistics from 400 ng/mL TNF-α. (**C**) Viability of 6499c4 EV and *Mgat5*-KO cells in vitro following exposure to varying concentrations of TNF-α or TRAIL. Statistics from 2 μg/mL TNF-α and 80 ng/mL TRAIL. (**D**) Viability of 6419c5 EV and *Mgat5*-KO cells in vitro following exposure to varying concentrations of TNF-α or TRAIL. Statistics from 2 μg/mL TNF-α and 500 ng/mL TRAIL. All previous data representative of at least 2 independent experiments. (**E**) Viability of 2838c3 EV and *Mgat5*-KO-A cells in vitro following exposure to varying concentrations of TNF-α with or without the pan-caspase inhibitor z-VAD (left) or the necroptosis inhibitor necrostatin-1 (right). Statistics from 16 ng/mL TNF-α. (**F**) Viability of 6499c4 EV and *Mgat5*-KO-A cells in vitro following exposure to varying concentrations of TRAIL with or without the pan-caspase inhibitor z-VAD (left) or the necroptosis inhibitor necrostatin-1 (right). Statistics from 10 ng/mL TRAIL. *P < 0.05; ****P < 0.0001.

T cells kill their targets through multiple mechanisms, including perforin, granzyme B, and induction of the extrinsic cell death pathway induced by several members of the TNF superfamily: TNF-α, TRAIL, and Fas ligand (FasL) (44–46). As noted previously, *Mgat5*-KO tumors exhibited a notable increase in the apoptosis marker CC3 compared with EV tumors. We therefore hypothesized that heightened sensitivity to apoptosis underlies the immune-mediated clearance of tumor cells. To test this possibility, we cultured T cell–inflamed *Mgat5*-KO or EV tumor cells with increasing concentrations of TNF-α, TRAIL, or FasL, and measured cell death. Cell viability staining revealed a significant increase in the death of T cell–inflamed *Mgat5*-KO cells (2838c3) compared with EV controls at low concentrations of TNF-α, whereas neither TRAIL nor FasL stimulation had this effect (Figure 4A). This increased cell death in KO cells upon stimulation with TNF-α was verified using Annexin V/PI staining (Supplemental Figure 9A). The non–T cell–inflamed line 6694c2 exhibited a similar increase in TNF-α sensitivity (Figure 4B), but no consistent increase in sensitivity to TRAIL or FasL (Supplemental Figure 9B).

We then expanded our analysis by evaluating the existing 6419c5 (non–T cell–inflamed) EV and *Mgat5*-KO lines and knocking out *Mgat5* in an additional T cell–inflamed line (6499c4) to assess sensitivity to TNF-related ligands (Supplemental Figure 9C). As with all other lines tested, 6499c4 *Mgat5*-KO cells had similar growth in vitro compared to EV controls (Supplemental Figure 9D). Surprisingly, the 6419c5 and 6499c4 lines were resistant to TNF-α–induced cell death (both EV and *Mgat5*-KO), but were instead sensitive to TRAIL-induced cell death, with *Mgat5*-deficient cells exhibiting greater sensitivity to these agents (Figure 4, C and D). Consistent with earlier observations, both lines were insensitive to FasL-induced cell death regardless of *Mgat5* status (Supplemental Figure 9, E and F). These results suggest that pancreatic tumor lines lacking *Mgat5* are more sensitive to the death-inducing activity of ligands belonging to the TNF superfamily, with some *Mgat5*-KO lines exhibiting enhanced sensitivity to TNF-α and others exhibiting enhanced sensitivity to TRAIL. This dichotomy may reflect the underlying sensitivities of the parental cell lines, as the 2838c3 and 6694c2 lines exhibited approximately 25%–50% cell death at the highest TNF-α concentrations, whereas the 6419c5 and 6499c4 lines were insensitive to TNF-α.

### Mgat5 loss increases susceptibility to both apoptosis and necroptosis.

TNF-α can have dichotomous effects on tumor growth and survival via canonical signaling through its 2 receptors, TNFR1 and TNFR2. Specifically, while TNFR1 signaling typically promotes cell death, stimulation of TNFR2 typically promotes cell survival and proliferation through the NF-κB signaling pathway (47, 48). To confirm that the enhanced sensitivity of TNF-α was mediated by TNFR1 signaling, we used CRISPR to KO TNFR1 in 2838c3 *Mgat5*-KO cells; as expected, double-KO cells were completely resistant to the death-inducing effects of TNF-α (Supplemental Figure 10A).

TNFR1-induced killing can occur through either apoptosis or necroptosis (49, 50). To distinguish between these possibilities, we treated 2838c3 *Mgat5*-KO or EV control cells with TNF-α and either the pan-caspase (apoptosis) inhibitor Z-VAD-FMK (z-VAD) (51) or the RIPK1 (necroptosis) inhibitor necrostatin-1 (52, 53). Inhibitors were validated using Western blotting to confirm knockdown of CC3 and cleaved caspase-8 (z-VAD) or phosphorylated RIPK1 (necrostatin-1) (Supplemental Figure 10, B and C). Whereas z-VAD marginally reversed the enhanced sensitivity to TNF-α caused by *Mgat5* deficiency, necrostatin-1 resulted in a near-complete rescue of the cell death phenotype (Figure 4E). The sensitivity to TNF-α of another *Mgat5*-KO line, 6694c2, was also reversed by necrostatin-1 (Supplemental Figure 10D).

Stimulation of the TRAIL receptor in cancer cells can also lead to cell death through either the apoptotic or necroptotic pathway (54, 55). We thus repeated the inhibitor experiments with the cell lines that were resistant to TNF-α but sensitive to TRAIL — 6499c4 and 6419c5. In these lines, necrostatin-1 had no effect; instead, the pan-caspase inhibitor z-VAD was able to fully reverse the increased sensitivity of *Mgat5*-KO cells to TRAIL-induced cell death (Figure 4F and Supplemental Figure 10E). These findings suggest that loss of *Mgat5* renders pancreatic tumor cells more susceptible to TNF-α and TRAIL by lowering the threshold for cells to undergo apoptotic and/or necroptotic cell death.

### Mgat5 glycans affect sensitivity to TNF-α–mediated cell death in lung and colorectal cancer cells.

To determine whether the increased sensitivity of *Mgat5*-deficient cells to TNF-α– or TRAIL-induced cell death is specific for PDAC or generalizable to other tumor types, we evaluated the effects of *Mgat5* KO in 4 non-PDAC cell lines: Lewis lung carcinoma (LLC) cells, MC38 colorectal cancer cells, B16-F10 melanoma cells, and Hep55 HCC cells. We used CRISPR to edit *Mgat5* in these cell lines and then used flow cytometry and cell sorting to isolate highly enriched populations of PHA-L^–^ (*Mgat5*-KO) cells.

**Figure 5:**
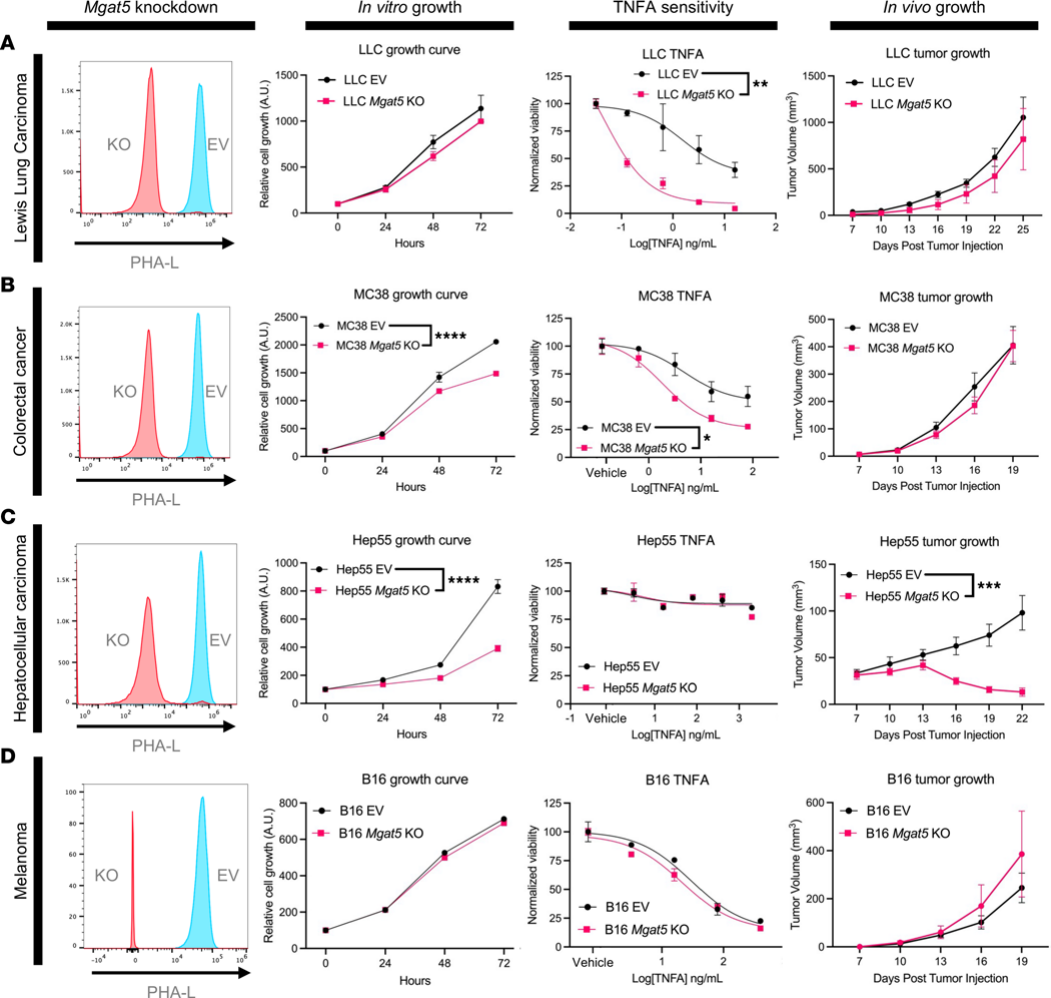
Mgat5 glycans protect some non–pancreatic cancer cells against TNF-α–mediated cell death. (**A**) Histogram of PHA-L binding, in vitro growth, in vitro cell death assays using TNF-α, and subcutaneous tumor growth of Hep55 EV and *Mgat5*-KO cells in *C57BL/6* mice. For in vitro growth assays, statistical analysis done using 2-way ANOVA with *n* = 3 replicates per data point for this and all further in vitro growth assays. In vivo growth representative of 2 independent experiments. Analysis for TNF-α death assay done using 2-way ANOVA at 400 ng/mL for this and all further TNF-α death assays at the specified concentration, and representative of 2 independent experiments. Analysis for tumor growth using 2-way ANOVA for this and all further tumor growth curves. Data represent mean ± SEM. (**B**) Histogram of PHA-L binding, in vitro growth, in vitro cell death assays using TNF-α, and subcutaneous tumor growth of LLC EV and *Mgat5*-KO cells in *C57BL/6* mice. Statistics for TNF-α death assay done at 3.2 ng/mL. (**C**) Histogram of PHA-L binding, in vitro growth, in vitro cell death assays using TNF-α, and subcutaneous tumor growth of MC38 EV and *Mgat5*-KO cells in *C57BL/6* mice. For in vitro growth assay, *n* = 6 replicates per data point. Statistics for TNF-α death assay done at 3.2 ng/mL. (**D**) Histogram of PHA-L binding, in vitro growth, in vitro cell death assays using TNF-α, and subcutaneous tumor growth of B16-F10 EV and *Mgat5*-KO cells in *C57BL/6* mice. Statistics for TNF-α death assay done at 80 ng/mL. *P < 0.05; **P < 0.01; ***P < 0.001; ****P < 0.0001.

Two of the 4 lines tested revealed increased sensitivity to TNF-α–mediated cell death, as observed in our PDAC lines. Similar to our PDAC cell lines, loss of *Mgat5* in LLC cells had no effect on in vitro growth at baseline, but significantly increased sensitivity to TNF-α–mediated cell death, with no effect on TRAIL- or FasL-induced cell death. Subcutaneous injection in vivo, however, revealed no tumor growth deficiencies in the *Mgat5*-KO line (Figure 5A and Supplemental Figure 11A). Loss of *Mgat5* in the colorectal cancer line MC38 resulted in a mild growth deficiency in vitro, increased sensitivity to TNF-α–induced cell death, but no effect on tumor growth in vivo (Figure 5B and Supplemental Figure 11B). The increased sensitivity found in the LLC and colorectal cancer lines confirms that the function of *Mgat5* in limiting the response of cells to TNF-α is not restricted to PDAC.

We next turned our attention to the HCC line Hep55. *Mgat5* expression has prognostic significance in HCC (16), and *Mgat5* influences multiple aspects of tumor biology (20, 23, 24, 56, 57). We found that *Mgat5* loss in Hep55 cells resulted in a significant growth defect in vitro without any increased sensitivity to the 3 extrinsic cell death ligands. Likewise, *Mgat5*-deficient Hep55 cells exhibited spontaneous tumor regressions in vivo, following the pattern of initial growth followed by rapid rejection, as seen in the PDAC lines (Figure 5C and Supplemental Figure 11C).

Finally, B16 cells lacking *Mgat5* exhibited ligand sensitivity and growth profiles (in vitro and in vivo) that were similar to EV controls (Figure 5D and Supplemental Figure 11D). These results emphasize the heterogeneity of glycosylation in different cell lines and tumor types, with the function of *Mgat5* in controlling sensitivity to TNF-α conserved in some non-PDAC cancers and its ability to promote tumor regression present in others.

### Loss of Mgat5 sensitizes tumors to immunotherapy.

**Figure 6:**
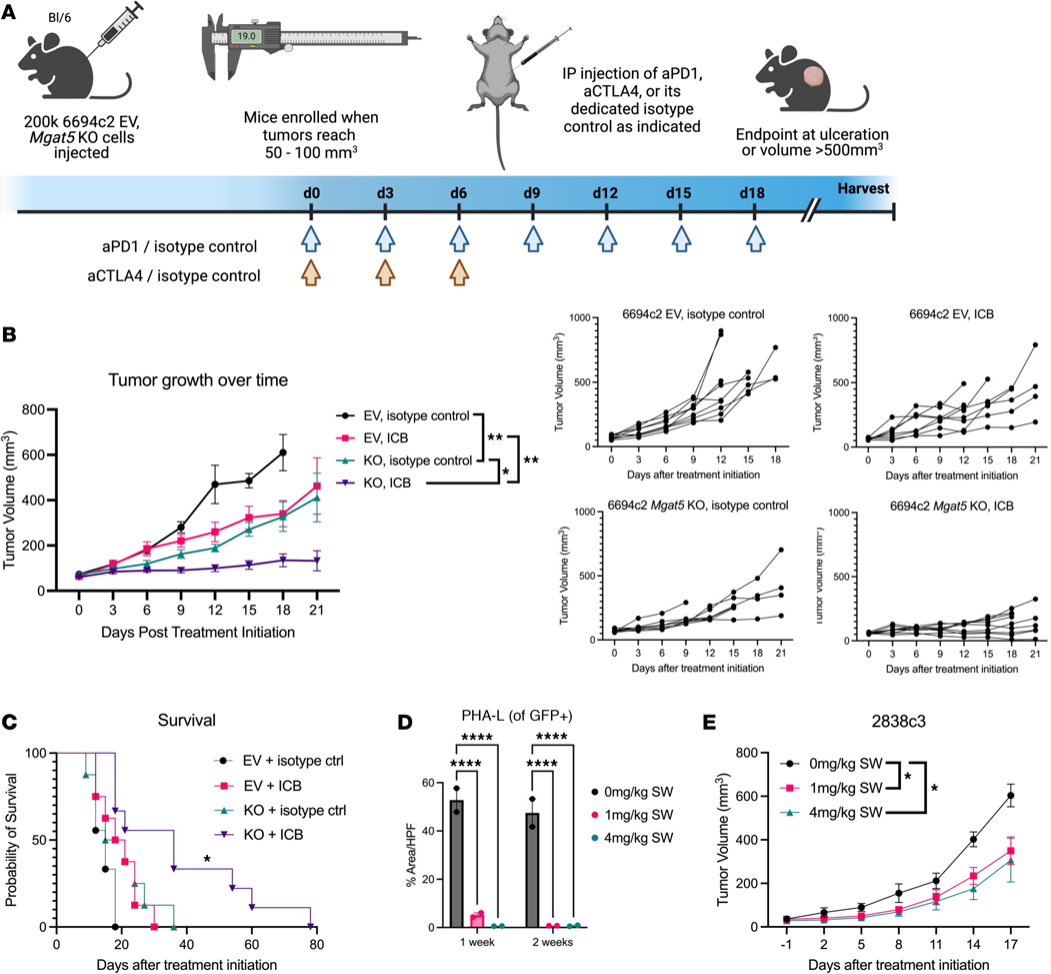
*Mgat5* loss augments the antitumor effects immune checkpoint blockade (ICB). (**A**) Schematic illustrating the ICB experimental design. (**B**) Growth of 6694c2 EV under isotype control (*n* = 9), EV treated with ICB (*n* = 8), *Mgat5*-KO under isotype control (*n* = 8), and *Mgat5*-KO treated with ICB (*n* = 9). Spaghetti plots showing the growth of individual tumors are presented on the right. Data represent mean ± SEM. Statistical analysis by 2-way ANOVA at day 9. Not shown is significance (****) of EV + isotype ctrl and KO + ICB groups. EV + isotype ctrl and EV + ICB groups are not significantly different (*P* = 0.4095). (**C**) Survival of mice from the experiment in **B**. Statistical analysis by log-rank (Mantel-Cox) test. (**D**) Microscopic evaluation of immunofluorescent PHA-L binding in 2838c3 WT tumors treated with 1 or 2 weeks of 0 mg/kg, 1 mg/kg, or 4 mg/kg swainsonine. Quantification of immunofluorescent staining for PHA-L using ImageJ. Two mice per condition per week (EV, KO) with with 3–5 HPF per tumor. Statistical analysis done using unpaired, 2-tailed Student’s *t* test. (**E**) Growth of 2838c3 WT subcutaneous tumors in mice treated with 0 mg/kg (*n* = 5), 1 mg/kg (*n* = 4), or 4 mg/kg (*n* = 5) of swainsonine administered once daily i.p. for 7 days. Mice with tumors sized 20–60mm^3^ were enrolled in treatment. Data represent mean ± SEM. Statistical analysis by 2-way ANOVA at day 14. *P < 0.05; **P < 0.01; ****P < 0.0001.

Given the finding that *Mgat5*-KO tumors were more susceptible to T cell–mediated antitumor activity, we hypothesized that *Mgat5* loss might sensitize tumors to T cell–directed immunotherapy. The increased number of PD-1^+^CD8^+^ T cells shown to be present in non–T cell–inflamed *Mgat5*-KO tumors as seen in Supplemental Figure 7B further suggests this may be an effective therapy, although no difference in PD-L1 expression on EV and *Mgat5*-KO tumor cells after stimulation with IFN-γ was observed in vitro (Supplemental Figure 12A). To test this, we used a non–T cell–inflamed line (6694c2), which is poorly responsive to combination ICB (40). We injected *C57BL/6* mice with either 6694c2 EV or *Mgat5*-KO cells and treated tumor-bearing animals with a combination of PD-1– and CTLA4-blocking antibodies when tumors reached 50–100 mm^3^ in volume (Figure 6A). Because tumors arise in only approximately 50% of mice injected with *Mgat5*-KO 6694c2 cells (Table 1), only those animals that developed tumors (i.e., those with the weakest *Mgat5*-KO phenotype) were used in the experiment. Despite this handicap, the addition of ICB treatment led to a significant decrease in tumor growth (Figure 6B) and improved survival (Figure 6C) of mice bearing *Mgat5*-KO tumors.

Given the immune effects of genetic loss of *Mgat5*, we reasoned that pharmacological inhibition of *Mgat5* might also have an antitumor effect in vivo. Swainsonine is an inhibitor of Golgi α-mannosidase II, which lies upstream of Mgat5 (58) and can efficiently inhibit the formation of PHA-L^+^ N-glycans in our system without inflicting cell death in vitro (Supplemental Figure 12, B and C). Although swainsonine showed initial promise as an anticancer agent, a phase II clinical trial failed to show efficacy in renal cell carcinoma (59–62). To determine whether pancreatic cancer is sensitive to pharmacological inhibition of Mgat5, we treated mice bearing T cell–inflamed 2838c3 WT tumors with either swainsonine (1 mg/kg or 4 mg/kg) or vehicle control for 2 weeks. IF of tumors taken after 1 week and 2 weeks of swainsonine treatment verified an effective reduction in PHA-L binding (Figure 6D). Both the 1 mg/kg and 4 mg/kg swainsonine dosages prompted a significant decrease in tumor growth compared with vehicle control–treated tumors (Figure 6E), indicating that pharmacologic inhibition of N-glycosylation can phenocopy disruption of the *Mgat5* gene.

## Discussion

The dysregulated glycosylation of tumor cells provides cancer cells with a powerful source of heterogeneity, allowing them to evade antitumor immunity. It is estimated that 50%–70% of human proteins are glycosylated, oftentimes at multiple sites through a series of glycan-modifying enzymes (63–65). In this study, we found that N-glycans formed by the Mgat5 glycosyltransferase confer robust protection against immune recognition and elimination of pancreatic cancer. Either genetic KO or pharmacological inhibition results in significantly decreased tumor growth in vivo, an effect that is T cell dependent and can essentially flip the immune phenotype of an immunosuppressive, non–T cell–inflamedline into a more CD8^+^-enriched microenvironment. Furthermore, the loss of Mgat5 glycans can sensitize cancer cells to either TNF-α– or TRAIL-mediated cell death through necroptotic and/or apoptotic pathways. Finally, the inability to produce Mgat5 glycans potentiates the activity of ICB, suggesting that targeting *Mgat5* may improve the efficacy of cancer immunotherapy.

Mgat5-dependent N-glycans play important roles in cell-cell and cell-matrix adhesion. Dysregulation of Mgat5 has previously been reported to promote cancer progression in various tumor types (66–68), and studies in PDAC have suggested that Mgat5 glycans interfere with the formation of the immunological synapse between T cells and cancer cells (69). Less well understood is the role of Mgat5 glycans in modulating tumor cell responses to pro-death stimuli. Previous work found that engagement of Mgat5-mediated N-glycans stimulates production of IFN-γ and TNF-α, in agreement with our study. Loss of *Mgat5* in breast cancer led to a decrease in tumor growth and an increase in the production of IFN-γ and TNF-α in splenocytes (18). In HCC, expression of IFN-γ and TNF-α by CD4^+^ T cells was found to be significantly diminished upon coculture with monocytic DCs and tumor cells overexpressing *Mgat5* (17). Moreover, Mgat5 protected HCC tumor cells from anoikis, a form of cell death following loss of cell-matrix interactions (23). Downregulation of *Mgat5* augmented the cytotoxic activity of all-*trans* retinoic acid, a finding that was later determined to be caused by the dysregulation of various pro- and antiapoptotic proteins, including Bad, Bcl-2, Bcl-xL, Bax, caspase-3, and p53 (56, 57). Decreased expression of Bcl-2 following downregulation of *Mgat5* has also been observed in nasopharyngeal carcinoma (20). Our study is thus consistent with the notion that Mgat5 regulates tumor cell sensitivity to extrinsic cell death stimuli, although context-dependent differences in the molecular details (e.g., induction of apoptosis vs. necroptosis across cell lines) highlight the complex and heterogeneous nature of Mgat5 activity.

The mechanism(s) by which loss of *Mgat5* lowers the threshold for cell death have yet to be delineated. TNFR1, TRAIL receptor, and Fas are all substrates for N-glycosylation (70–73), and the loss of these glycans could enhance their death signaling activity. Alternatively, the function or expression of downstream pro- and antiapoptotic and/or necroptotic proteins could change following the loss of Mgat5 glycans. Given the complex nature of protein glycosylation, it is likely that the regulation of cell death thresholds by Mgat5 glycans is multifactorial.

An alternative explanation for the robust T cell response to *Mgat5*-deficient tumors is that *Mgat5* loss prompts the formation of potent neoepitopes; however, several lines of evidence argue against this possibility. First, immunological memory (resulting in the rejection of tumor cells upon rechallenge) depends on a live T cell–tumor cell interaction, suggesting that new antigens formed as a consequence of the *Mgat5* KO (if any) are insufficiently strong on their own to generate a durable immune response. Second, any *Mgat5*-KO–related neoantigens strong enough to result in T cell–mediated tumor clearance would be expected to reshape the T cell receptor repertoire. However, our Vβ repertoire analysis did not reveal the expansion of new T cell receptor subsets in *Mgat5*-KO tumors; rather, T cell subsets that were already expanded in response to *Mgat5*-WT tumors underwent further expansion in *Mgat5*-KO tumors. While it remains possible that *Mgat5* loss results in the formation of some new antigens*,* we found no evidence that antigens play a functional role in the enhanced immune response. Rather, our findings suggest that *Mgat5* glycans impede the activity and function of tumor-specific T cells and that their elimination enhances immunogenicity in an antigen-agnostic manner.

Finally, our study provides support for *Mgat5* as a target for clinical translation. Although the immune nature of our *Mgat5*-KO phenotype precluded use of human PDAC models in vivo, promising work by Greco et al. determined that *MGAT5* KO in human PDAC cells resulted in improved targeting by CAR T cells (69). *Mgat5* deletion in a non–T cell–inflamed (“cold”) PDAC tumor line synergized with ICB to significantly slow tumor growth, improve survival, and promote rare tumor regressions. Moreover, treatment with swainsonine, a broad-spectrum inhibitor of N*-*glycosylation, inhibited the growth of T cell–inflamed tumors. Previous clinical trials with swainsonine did not yield clinically meaningful responses in other tumor types. However, newer, more specific inhibitors of Mgat5 enzymatic activity are under development (74), and the activity of such agents in combination with immunotherapy merits further study.

## Methods

### Sex as a biological variable

Both male and female mice were used in this study and no differences were observed between the sexes.

### Animals

Mice were purchased from The Jackson Laboratory, bred and maintained in specific pathogen–free facilities at the University of Pennsylvania, and used in randomized unblinded experiments. Mouse strains used include *C57BL/6* (stock 000664), *NOD/SCID* (stock 006848), *Batf3*^–/–^ (stock 013755), and *muMT*^–^ (stock 002288). Mice were used at 6–8 weeks of age.

### Bioinformatics analysis

Differential expression of *Mgat5* in human PDAC and normal pancreas samples analyzed with the GEPIA2 server created by Tang et al. (75). Data sets used in the analysis are from the TCGA Research Network (https://www.cancer.gov/tcga) and the Genotype-Tissue Expression (GTEx) Project (https://gtexportal.org/home/).

### Tumor cell lines

Murine T cell–inflamed (2838c3, 6499c4) and non–T cell–inflamed (6419c5, 6694c2) clones were derived from the *KPC/Y* model of pancreatic cancer, as described in Li et al. (40), at the University of Pennsylvania. *Mgat5* KOs were generated using the lentiCRISPRv2 vector (Addgene plasmid 52961) with sgRNA forward (CACCGGCTGTCATGACACCAGCGTA) and reverse (AAACTACGCTGGTGTCATGACAGCC) primers. TNFR1 KOs were generated using the same vector with sgRNAs CACCGGTTTAATGTGCCGATATCCC and CACCGAGACCTAGCAAGATAACCAG. The Hep55 line was provided by the Haldar lab, and the LLC, MC38, and B16-F10 lines provided by the Vonderheide lab, both from the University of Pennsylvania. All lines were originally sourced from ATCC. The lentiCRISPRv2 KO or EV was packaged with VSVG and PAX2 and transfected into HEK293T cells for lentivirus production. Tumor cells were infected with lentivirus for 24 hours, with puromycin added for selection at 48 hours after transfection. After 2 weeks of puromycin selection, clonal KO lines were derived using limiting dilution followed by flow cytometry for PHA-L binding. Cell lines were regularly assessed for *Mycoplasma* using the MycoAlert Mycoplasma Detection Kit (Lonza, LT07-0318). All tumor lines were cultured in DMEM with 10% FBS and 1% penicillin-streptomycin (Pen/Strep) (Invitrogen, MT30-002-Cl) and maintained at 37°C. Passage numbers were kept below 15.

### Cell growth assays

Cell growth in vitro was assessed via the plating of 500–1500 cells of the desired tumor lines in growth media in four 96-well tissue culture–treated plates. On the following day and each day thereafter, the number of viable cells was assessed using the CellTiter-Glo 2.0 Cell Viability Assay (Promega, G9241). Relative cell viability was calculated after normalization to the first day’s average reading. High-glucose media contained 4 mM L-glutamine (Corning, 25-005-Cl), 25 mM D-(+)-glucose (Sigma-Aldrich, G8769), 10% FBS, and 1% Pen/Strep. Low-glucose medium contained 4 mM L-glutamine, 2.5 mM D-(+)-glucose, 10% FBS, and 1% Pen/Strep. Low-FBS medium was made using DMEM with 1% FBS and 1% Pen/Strep.

### Quantitative PCR analysis

RNA was isolated from adherent tumor cells using NucleoSpin RNA (Macherey-Nagel, 740955.50) and reverse transcribed to cDNA with the High-Capacity cDNA Reverse Transcription Kit (Applied Biosystems, 4368813). The SSo Advanced Universal SYBR Green Supermix (Bio-Rad, 172-5274) was used for qPCR with normalization to *Tbp*. Primers used for *Mgat5* were TACGGGAGCAGATCCTTGAC (forward) and TGACCAGATTGTCCACCTTTGA (reverse). Primers for the control *Tbp* were CCACTCACAGACTCTCACAAC (forward) and CTGCGGTACAATCCCAGAACT (reverse). The QuantStudio 6 Real-Time PCR System, 384-well (Applied Biosystems, A43182) was used for analysis.

### Western blotting

Adherent tumor samples were grown to 70% confluence, washed twice with cold PBS, and lysed using 1× RIPA buffer (diluted from 10× RIPA; Cell Signaling Technology, 9806) with protease inhibitory cocktail (Sigma-Aldrich, CO-RO). Samples were centrifuged at 16,100*g* for 20 minutes in 4°C to remove insoluble materials. Protein content was determined using the Pierce BCA Protein Assay Kit (Thermo Fisher Scientific, 23227). Samples were diluted in 4× Laemmli Sample Buffer (Bio-Rad, 1610747) with β-mercaptoethanol (Sigma-Aldrich, M7154), denatured at 100°C for 8 minutes, and 30 μg of each sample was resolved using Bis-Tris 4%–12% gels (Invitrogen, NP0335BOX). The Precision Plus Protein Dual Color Standard (Bio-Rad, 1610374) used as a protein ladder. Separated samples were transferred to Immobilon-FL PVDF membranes (Millipore, IPFL00010) and probed with desired primary and secondary antibodies. Analysis was done using the LI-COR Odyssey imaging system and the ImageStudio software. Primary antibodies included those against α-tubulin (Cell Signaling Technology, 3873S), CC3 (Cell Signaling Technology, 9661S), cleaved caspase-8 (Cell Signaling Technology, 8592S), and phosphorylated RIPK1 (Cell Signaling Technology, 31122S). Secondary antibodies included IRDye 680 RD goat anti–mouse IgG (LI-COR, 926-68070) and IRDye 800CW goat anti–rabbit IgG (LI-COR, 926-32211).

### Tumor cell implantation

Murine cell lines were grown to 70% confluence and dissociated using incubation with 0.25% trypsin (Gibco) at 37°C for 1–5 minutes as appropriate. Cells were then washed with ice-cold PBS twice and total cell number acquired using the Countess 3 Automated Cell Counter (Thermo Fisher Scientific). Single-cell suspensions of 2 × 10^5^ to 2 × 10^6^ cells per 100 μL injection volume were prepared in DMEM. Cell suspensions were kept on ice until subcutaneous injection into mice ages 6–8 weeks old. Orthotopic injections with 1 × 10^5^ cells, prepared as above, were implanted directly into the pancreas. Measurements of tumor length and width were conducted every 3 days with electronic calipers starting 1 week after tumor injection, and volumes calculated using the formula (length × width^2^)/2.

### In vivo treatments

#### T cell and NK cell depletion.

Mice were injected intraperitoneally with 200 μL of either 200 μg anti-NK1.1 (BioXcell, BE0036), a mixture of 200 μg each of anti-CD4 (BioXcell, BE0003-1, clone GK1.5) and anti-CD8 (BioXcell, BE0061, clone 2.43), or the isotype control (BioXcell, BE0085, clone C1.18.4 for NK depletion; BioXcell, BE0090, clone LTF-2 for CD4/CD8). Suspensions were prepared in PBS. Intraperitoneal injection was performed in the lower quadrant, with the right and left side switched each injection day. NK1.1 depletion was begun 3 days prior to tumor cell injection, while CD4^+^/CD8^+^ cell depletion was begun on the same day as tumor cell injection. Depletion was continued every 3 days until experiment end.

#### ICB.

For ICB experiments, mice were enrolled when tumors reached 50–100 mm^3^ volume. Mice were injected intraperitoneally with 200 μg anti–PD-1 (BioXcell, BE0146, clone RMP 1-14) every 3 days for 7 total doses and 200 μg anti-CTLA4 (BioXcell, BE0131, clone 9H10) every 3 days for 3 total doses or the isotype control rat IgG2a mAb (clone 2A3, BioXcell). Suspensions were prepared to a total volume of 200 μL in PBS. Intraperitoneal injection was performed in the lower quadrant, with the right and left side switched each injection day.

#### Swainsonine.

Swainsonine (Cayman Chemical, 16860) was dissolved in DMSO to 10 mg/mL and then diluted in PBS to the desired concentration. The amount of DMSO in each dilution was equalized to ensure phenotypes were not due to DMSO injection. Mice were weighed and the desired dose of swainsonine (0, 1, and 4 mg/kg) was injected intraperitoneally every day for 14 days.

### Flow cytometry of cultured cells

Adherent cells in culture were dissociated using TrypLE Express (Gibco, 12605010), washed twice with cold PBS, and incubated with desired antibodies for 20–30 minutes at 4°C in flow buffer. Cells were then washed with PBS and incubated with secondary antibody as desired using the same conditions. Flow cytometry was performed on the Attune NxT (Thermo Fisher Scientific) and analyzed with FlowJo software (Tree Star). Reagents used for flow cytometry of cultured cells included biotinylated PHA-L (Vector Laboratories, B-1115-2), streptavidin APC (BioLegend, 405207), anti–PD-L1 PE/Cy7 antibody (BioLegend, 124314), and DAPI (Invitrogen, D21490).

### Flow cytometry of murine tumor samples

Mice were euthanized and subcutaneous tumors dissected into cold PBS. Tumors were minced to small size and incubated in DMEM with 1 mg/mL collagenase IV in DMEM and 3% DNase I (Sigma-Aldrich) to digest tumors. Tubes were inverted vigorously every 10 minutes for a total of 40 minutes of incubation at 37°C. Tumor digest was passed through a 70-μm cell strainer (MedSupplyPartners, CT-229482) to obtain single-cell suspensions and washed twice with PBS before staining with desired antibodies for 30 minutes at 4°C in flow buffer. Cells were fixed using 2% paraformaldehyde incubation for 15 minutes at 4°C. Flow cytometry was performed on the LSR II flow cytometer (BD Biosciences). Antibodies used in the staining of immune populations were as follows: CD279 (PD-1) FITC (BioLegend, 135214); CD335 (NKp46) PE (BioLegend, 137604); CD103 PE/Dazzle 594 (BioLegend, 121430); CD3 PE/Cy5 (BioLegend, 100300); CD8a PE/Cy7 (BioLegend, 100722); Ly-6G V450 (BD Biosciences, 560603); CD44 APC (BioLegend, 103012); CD45 AF700 (BioLegend, 103128); F4/80 APC/Cy7 (BioLegend, 123118); CD11b PerCP-Cy5.5 (BD Biosciences, 561227); Ly-6C BV570 (BioLegend, 128030); and CD4 BV650 (BioLegend, 100546). Exclusion of dead cells was accomplished using the LIVE/DEAD Fixeable Aqua Dead Cell Stain Kit (Thermo Fisher Scientific, L-34966).

### Flow cytometry of draining lymph nodes

Mice were euthanized and inguinal and axillary lymph nodes from the tumor-bearing side of the mouse isolated into cold PBS. Lymph nodes were minced to small size and incubated with 1 mg/mL collagenase IV in DMEM for 20 minutes at 37°C. Cells were then passed through the cell strainer cap of a FACS tube to obtain a single-cell suspension, washed with PBS, and moved to a 96-well V-bottom plate. Cells were stimulated for 4–5 hours in 150 μL in the dark at 37°C in the following stimulation media: PMA (50 ng/mL; Sigma-Aldrich, P-8139), ionomycin (500 ng/mL; Sigma-Aldrich, I-0634), GolgiPlug protein transport inhibitor (1:1000; BD Biosciences, 555029), and CD107a FITC (BioLegend, 121606) in tumor growth medium (DMEM with 10% FBS and 1.5% gentamicin). After incubation, cells were washed and stained with the extracellular flow panel antibodies CD44 BV605 (BD Biosciences, 563058), CD4 BV711 (BioLegend, 100550), CD8 BV785 (BioLegend, 100750), PD-1 APC (BioLegend, 109111), CD62L APC/Cy7 (BioLegend, 104428), CD45 PE/Dazzle 594 (BioLegend, 103146), and CD3 PE/Cy5 (BioLegend, 100310). After washing, cells were fixed using Fixation/Permeabilization solution (BD Biosciences, 554714) for 20 minutes at 4°C. Cells were washed twice in Perm/Wash buffer and then resuspended with the intracellular flow panel containing antibodies against IFN-γ PerCP/Cy5.5 (BioLegend, 505822), granzyme B Pacific Blue (BioLegend, 515408), FOXP3 PE (BD Biosciences, 560408), TNF-α PE/Cy7 (BioLegend, 506324), and Ki67 AF700 (BD Biosciences, 561277). Intracellular staining proceeded overnight in the dark at 4°C. Flow cytometry was performed on the LSR II flow cytometer (BD Biosciences).

### TCR repertoire analysis

Spleens were dissected into cold PBS and placed in a 70-μm cell strainer (MedSupplyPartners, CT-229482) in a 60-mm cell culture dish with PBS and ground using the knob of a 3 mL syringe to form a single-cell suspension. Red blood cells were lysed using RBC lysis buffer (G-Biosciences, 786-672) and the resulting suspension washed with PBS. Cells were counted and aliquots of 1 million splenocytes were moved to 96-well plates for analysis. Cells were stained with the Anti-Mouse TCR Vβ Screening Panel (BD Pharmingen, 557004) along with CD45 AF700 (BioLegend, 103128), CD3 PerCP/Cy5.5 (BioLegend, 100328), CD4 BV605 (BioLegend, 100547), CD8 PE/Cy7 (BioLegend, 100722), and LIVE/DEAD Fixeable Aqua Dead Cell Stain Kit (Thermo Fisher Scientific, L-34966) and analyzed on the Attune NxT (Thermo Fisher Scientific).

### Cell death assay

To examine sensitivity to different extrinsic cell death pathways, 2 × 10^3^ cells of the desired cell lines were plated in 96-well plates. The following day, ligands and adjuvants were added in varying concentrations as indicated in the text. For TNF-α–induced cell death, adjuvants of 0.2 μg/mL IFN-γ (Peprotech, 315-05) and 1 μg/mL cycloheximide were used in addition to TNF-α (Biotechne, 410-MT). For TRAIL (Biotechne, 1121-TL) and Fas ligand (Biotechne, 6128-SA), only 1 μg/mL cycloheximide was used as an adjuvant. After 48 hours of culturing, the viability was read using the CellTiter-Glo 2.0 Cell Viability Assay (Promega, G9241). Relative cell viability was calculated using adjuvant-alone growth of each cell line as the normalization parameter.

### Tumor cell lysis

Tumor cells were grown to 70% confluence and dissociated using 0.25% trypsin (Gibco). For cell lysis using sonication, samples were suspended in DMEM, counted, and adjusted to 2 × 10^5^ cells per 100 μL. They were then sonicated at 30 seconds on/30 seconds off for 5 cycles. Cells were stained with trypan blue and assessed for viability to ensure cell death before subcutaneous injection into mice as described above. For cell lysis using radiation, cultured cells had 10 Gy applied using the Cs-137 Gammacell irradiator (Nordion) and allowed to incubate for 72 hours prior to injection.

### H&E staining, IF staining, and analysis

Tumors were dissected into cold PBS, washed 3 times with PBS, and fixed in zinc-formalin for 24 hours at 4°C. Tissues were dehydrated using successive washes of increasing ethanol percentages from 70% to 100%, and then embedded in paraffin. For staining, tumor sections were deparaffinized, rehydrated, and blocked with a solution of 5% donkey serum and 0.3% Triton X-100 in PBS while shaking at room temperature for 1 hour. Primary antibodies were prepared in PBS with 5% donkey serum and 0.1% Tween 20 and incubated overnight at 4°C. Slides were then washed and incubated with secondary antibodies at room temperature for 1 hour. The Olympus IX71 inverted microscope and DP71 camera were used for imaging. Images then quantified in ImageJ (NIH) for percentage area (on GFP^+^ as appropriate) and values averaged per tumor, with 3–5 high-power fields (HPFs) taken per tumor. The averaged value per tumor is reported in the figures.

Primary antibodies used for IF included those against CC3 (Cell Signaling Technology, 9661S), CD8a (Cell Signaling Technology, 98941S), biotinylated PHA-L (Vector Laboratories, B-1115-2), GFP (Abcam, 13970), granzyme B (Cell Signaling Technology, 46890S), FOXP3 (Invitrogen, 14-5773-80), and aSMA (Sigma-Aldrich, A2547). Secondary antibodies used include donkey anti–rabbit IgG AF488 (Invitrogen, A-21206), donkey anti–chicken IgG AF647 (Jackson ImmunoResearch, 703-605-155), donkey anti–rat IgG AF594 (Invitrogen, A-21209), and streptavidin AF488 (Thermo Fisher Scientific, S32354).

### Statistics

For cell death studies, values were normalized to control, concentrations were converted to log values, and log(inhibitor) versus response (3 parameters) test was used to create lines of nonlinear fit. Two-tailed Students *t* test and 1-way and 2-way ANOVA were used for comparison as appropriate. For experiments where 1 measurement was taken per animal and multiple animals per treatment group, a 1-way fixed-effects model was used. For experiments with repeated measures over time, a 2-way mixed effects model with estimation of within- and between-animal variability was used. Tukey’s multiple-comparison test was used as a post hoc method to compare treatments. In the 2-way mixed-effects model, only the comparison on the last day is presented. Statistical analysis and graph generation were performed using GraphPad Prism version 10.0.3.

### Study approval

The University of Pennsylvania IACUC approved all animal experiments.

### Data availability

The Supporting Data Values file provides values for all graphs and means reported.

## Author contributions

EEH, BZS, SLC, and KEW designed the research study. EEH, REF, SLC, JCM, MIO, SBK, BMK, IKK, and WPV conducted experiments and analyzed the data. SLC provided the KO vector and 2838c3 *Mgat5*-KO-A cell line. EEH wrote the manuscript. BZA, REF, SBK, IKK, and BMK edited the manuscript.

## Supplementary Material

Supplemental data

Supporting data values

## Figures and Tables

**Table 1:**
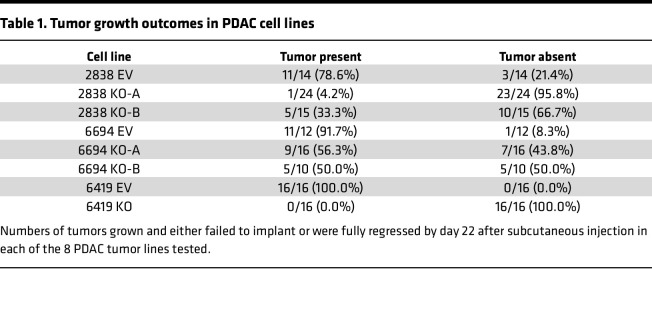
Tumor growth outcomes in PDAC cell lines

## Version 1 06/24/2024

Electronic publication
