# Supplementary material for: N-glycosylation by Mgat5 imposes a targetable constraint on immune-mediated tumor clearance
Source: JCI Insight. 2024 May 23;9(12):e178804. doi: 10.1172/jci.insight.178804 (PMC11383181; doi:10.1172/jci.insight.178804)
Supplement: Supplemental data [file jciinsight-9-178804-s166.pdf]

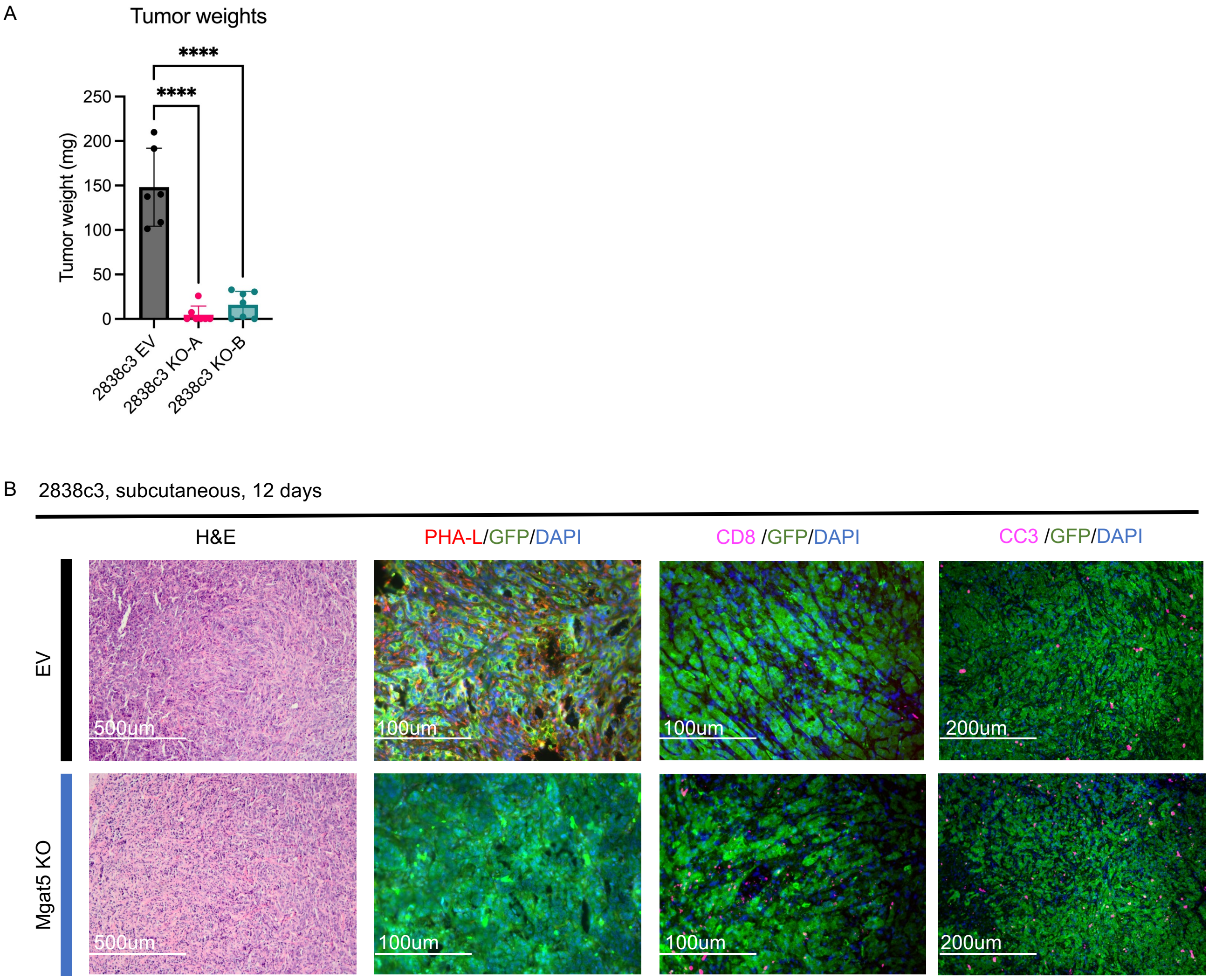

Figure S1: Histologic and immunofluorescent analysis of T cell-inflamed EV and Mgat5 KO tumors. A, Weights of 2838c3 EV, Mgat5 KO-A, and Mgat5 KO-B subcutaneous tumors in *C57BL/6* mice. Data represent mean ± SEM (*n* = 6-7 mice/group). Statistical analysis done using one-way ANOVA. **B,** Microscopic evaluation of 2838c3 EV and Mgat5 KO tumors fixed at twelve days post injection by H&E or immunofluorescence PHA-L, CD8, or CC3.

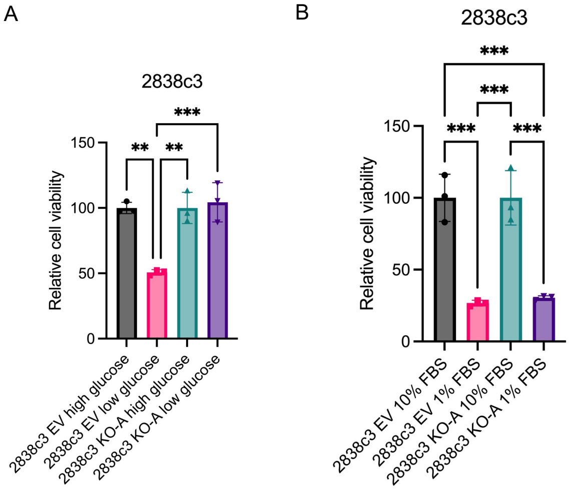

Figure S2: Growth of T cell-inflamed lines in low glucose and FBS. A, Relative cell viability by CellTiter Glo analysis of 2838c3 EV and Mgat5 KO cells grown in high glucose (25mM) or low glucose (2.5mM). Viability is normalized to high glucose condition. Statistical analysis by one-way ANOVA. Error bars are SD. **B,** Relative cell viability by CellTiter Glo analysis of 2838c3 EV and Mgat5 KO cells grown in 10% FBS (normal) or 1% FBS. Viability is normalized to 10% FBS condition. Statistical analysis by one-way ANOVA. Error bars are SD.

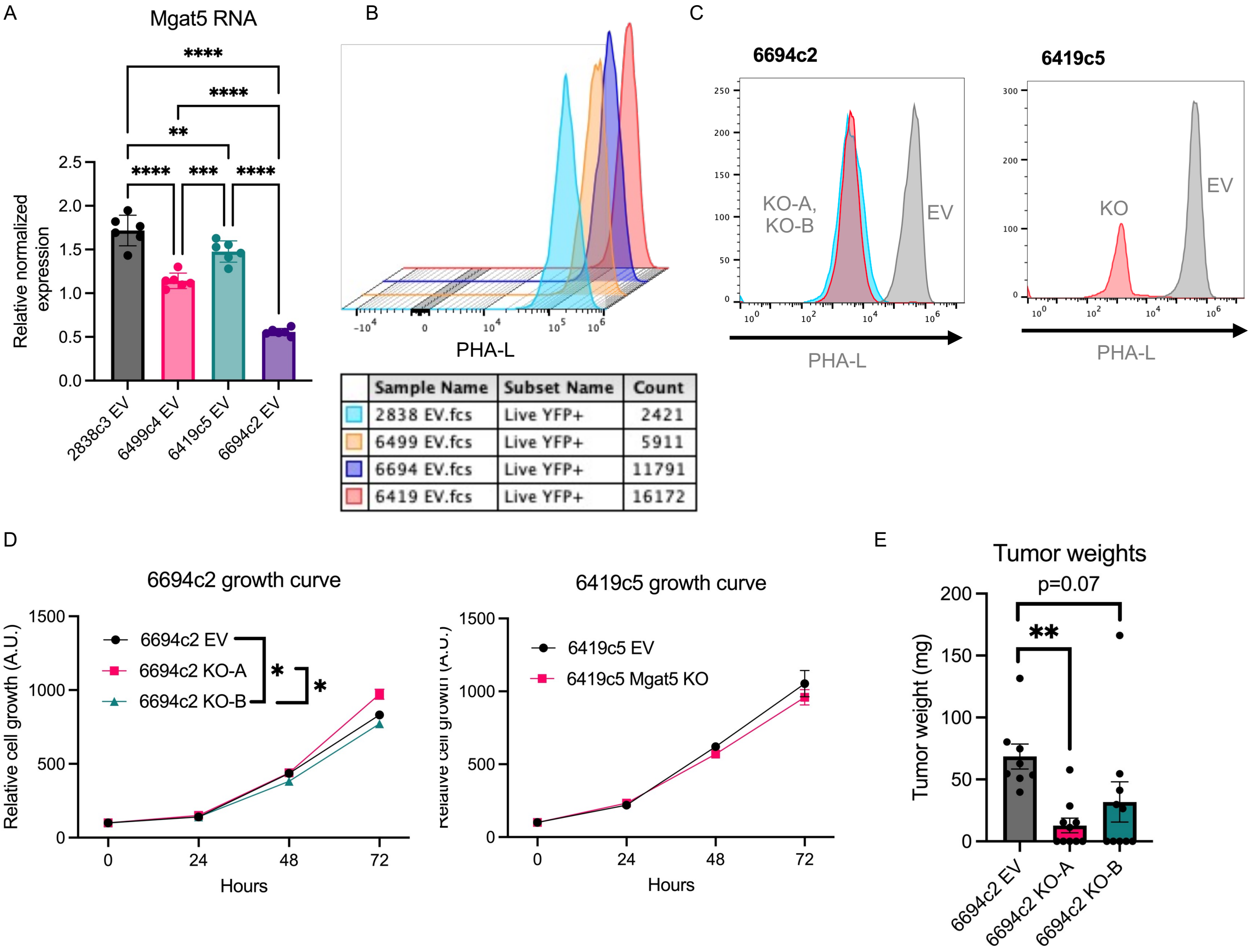

Figure S3: Characterizing Mgat5 KO in multiple PDAC lines. A, qPCR analysis of Mgat5 RNA content in the 2838c3, 6499c4, 6694c2, and 6419c5 EV lines. Statistical analysis by one-way ANOVA. Error bars are SD. **B,** Histogram of PHA-L binding to 2838c3, 6499c4, 6694c2, 6419c5 EV lines, normalized to mode. **C,** Histogram of PHA-L binding to non-T cell-inflamed (6694c2, 6419c5) EV and Mgat5 KO cell lines. **D,** Relative growth of non-T cell-inflamed (6694c2, 6419c5) EV and Mgat5 KO cell lines *in vitro*. Statistical analysis done using two-way ANOVA with *n* = 3 replicates per data point. **E,** Weights of 6694c2 EV, Mgat5 KO-A, and Mgat5 KO-B subcutaneous tumors in *C57BL/6* mice. Tumor weights were obtained from dissected tumors at day 22. Data represent mean ± SEM (*n* = 8-10 mice/group). Statistical analysis done using one-way ANOVA.

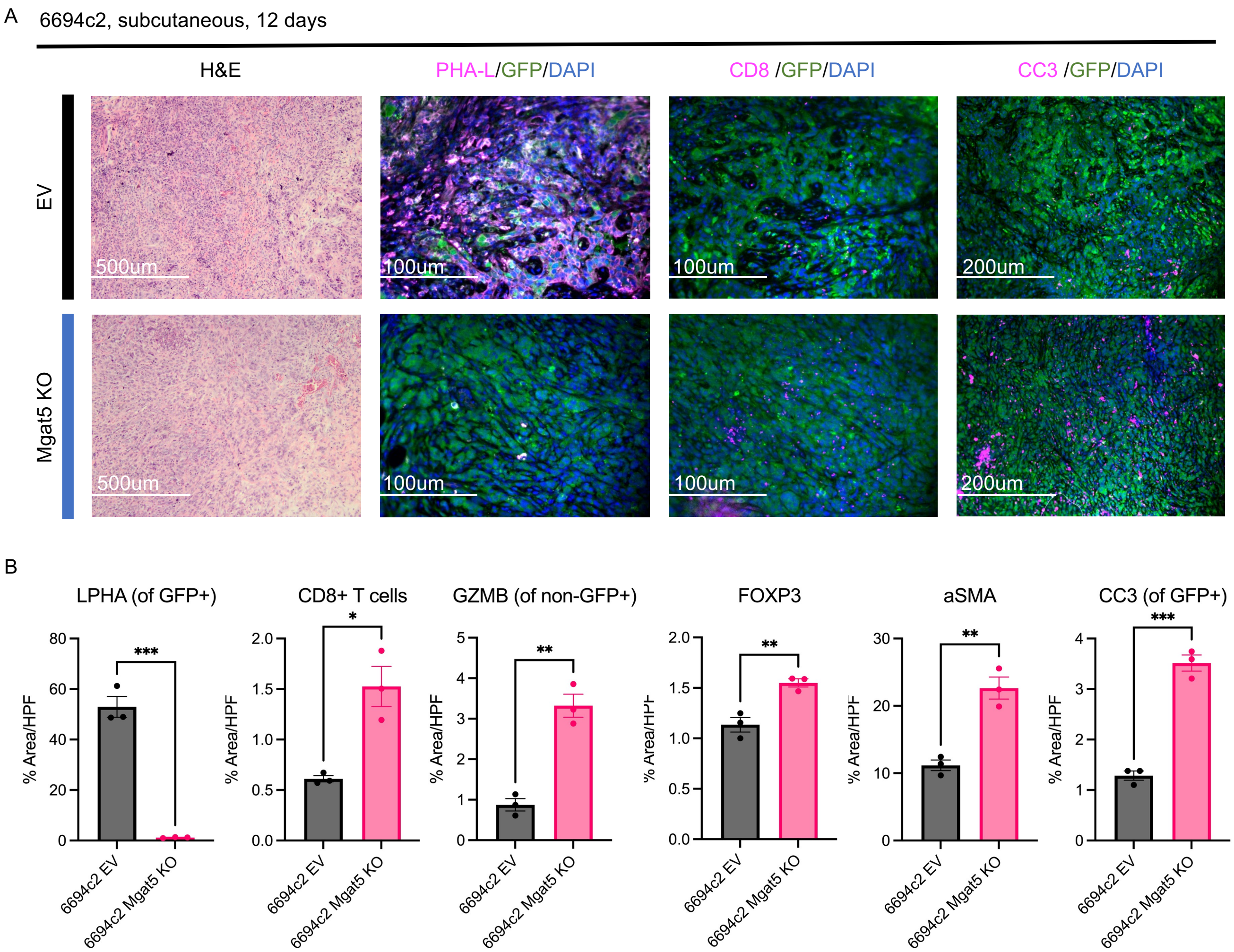

Figure S4: Histologic and immunofluorescent analysis of non-T cell-inflamed EV and Mgat5 KO tumors. A, Microscopic evaluation of 6694c2 EV and Mgat5 KO tumors fixed at twelve days post injection by H&E (i) or immunofluorescence PHA-L (ii), CD8 (iii), or CC3 (iv). **B,** Quantification of immunofluorescent staining in 6694c2 tumors for PHA-L, CD8 T cells, Granzyme B (GZMB), FOXP3, aSMA, and cleaved caspase 3 (CC3) by percent area per high-power field (HPF) using ImageJ. Three mice per condition (EV, KO) with with 3-5 HPF per tumor. Statistical analysis by unpaired Student's *t* test.

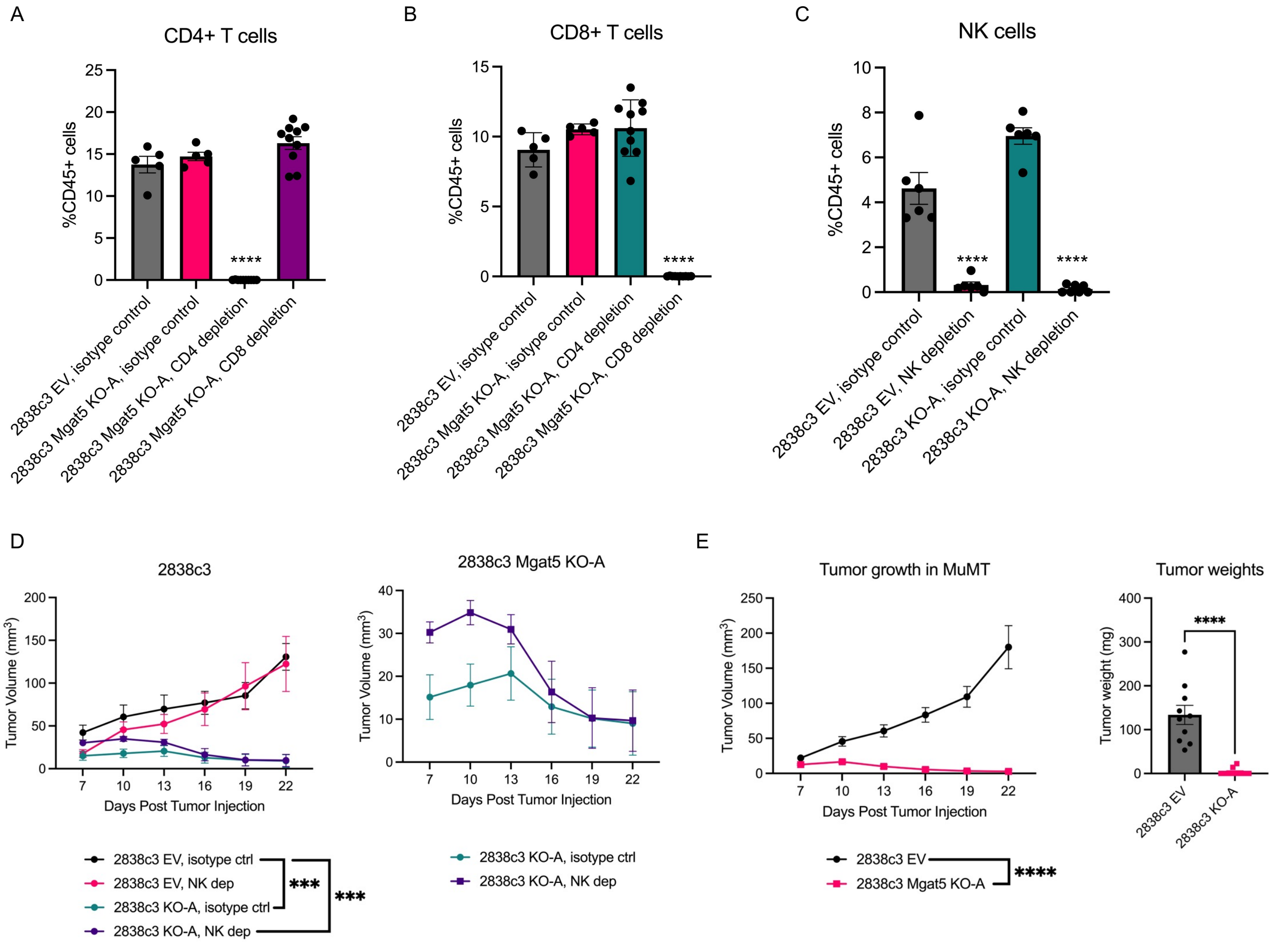

Figure S5: Immune involvement in Mgat5 KO tumor clearance. A, Confirmation of depletion of CD4+ T cells via flow of the spleen at experiment end. Statistics using one-way ANOVA for this and all depletion validation. **B,** Confirmation of depletion of CD8+ T cells via flow of the spleen at experiment end. **C,** Confirmation of depletion of NK cells via flow of the liver at experiment end. **D,** Subcutaneous tumor growth over time of 2838c3 EV under isotype control (n=6) or NK depletion (n=6), and Mgat5 KO-A tumors under isotype control (n=6) or NK depletion (n=7). Left, all four lines. Right, Mgat5 KO lines under isotype and NK cell depletion alone. Statistical analysis done using two-way ANOVA. Data represent mean ± SEM. **E,** Subcutaneous tumor growth over time of 2838c3 EV (n=10) and Mgat5 KO-A (n=10) tumors in MuMT mice lacking B cells. Data represent mean ± SEM. Statistical analysis using two-way ANOVA.

A

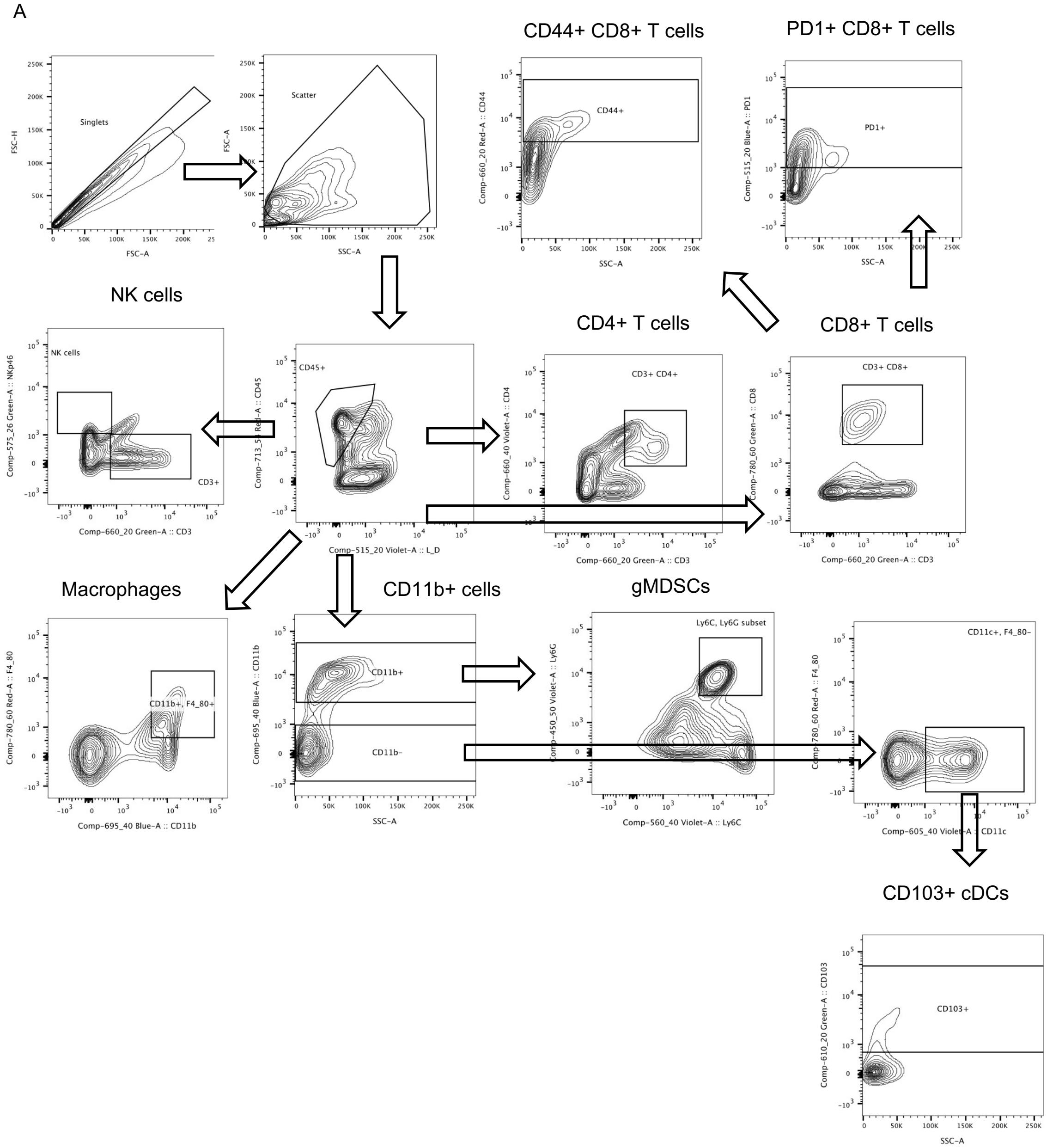

Figure S6: Flow cytometry gating for immune phenotyping of Mgat5 KO tumor microenvironment. A, Gating schema for immune phenotyping of tumor microenvironment to define CD4+ T cells, CD8+ T cells, CD44+ CD8+ T cells, PD1+ CD8+ T cells, NK cells, CD11b+ myeloid cells, macrophages, gMDSCs, and CD103+ cDCs.

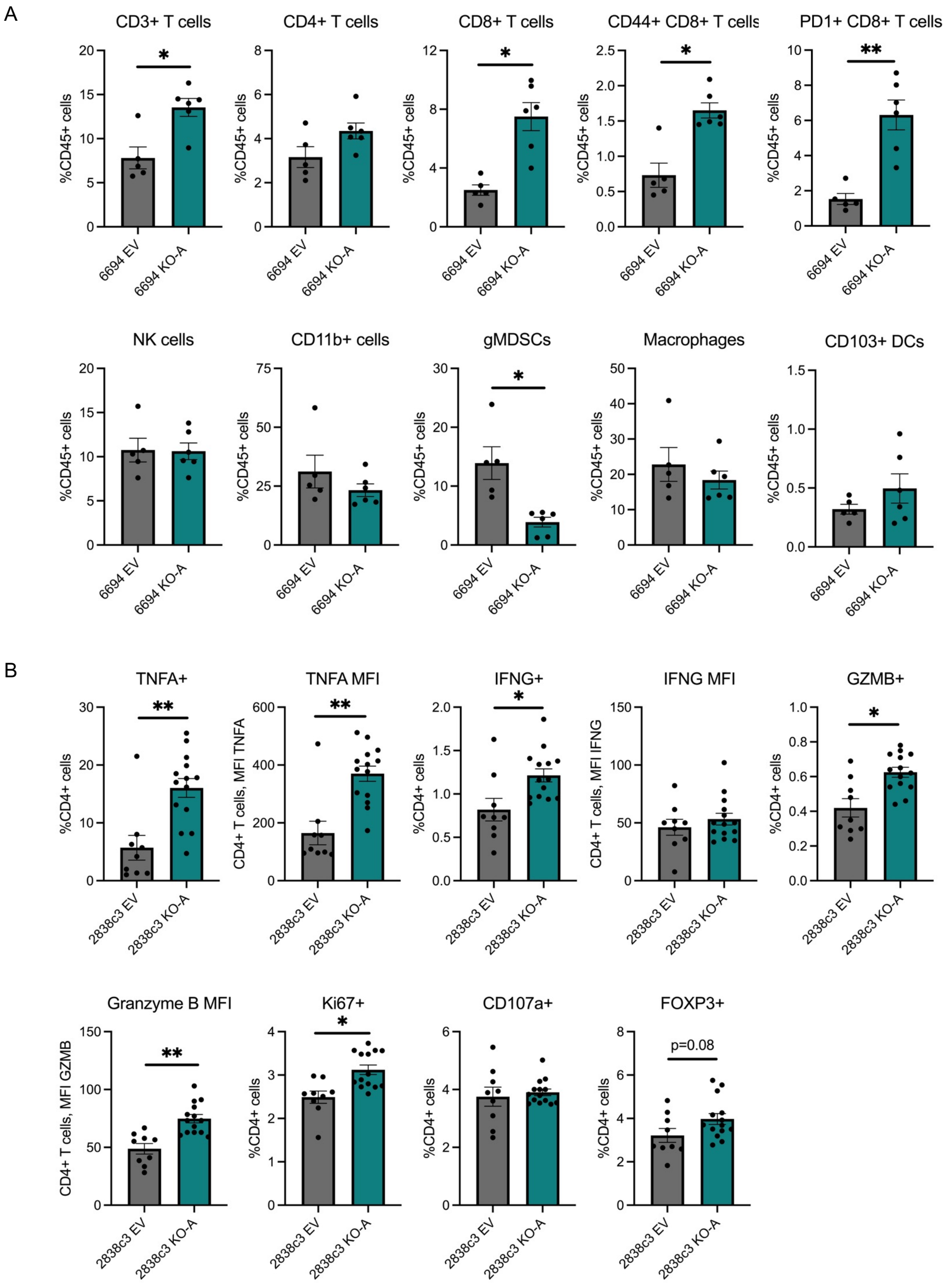

Figure S7: Analysis of the tumor immune microenvironment and T cell cytotoxicity in EV and Mgat5 KO tumors. A, Flow cytometry analysis of the indicated immune cell subsets in 6694c2 EV (*n* = 5) and Mgat5 KO-A tumors (*n* = 6) harvested at d22 post subcutaneous injection. Data represent mean ± SEM. Statistical analysis using unpaired Student's *t* test. Data representative of two independent experiments. **B,** Flow cytometry analysis of T cell cytotoxicity in draining lymph nodes (inguinal, axillary) from 6694c2 EV and Mgat5 KO subcutaneous flank tumor at d12 post injection. Data represent mean ± SEM. Statistical analysis using unpaired Student's t test. Data representative of two independent experiments.

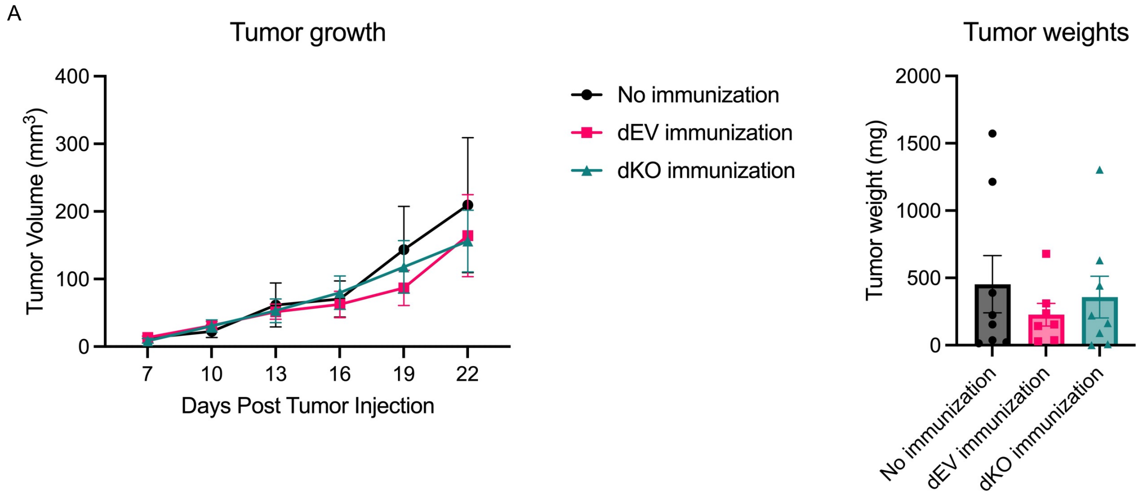

Figure S8: Challenge experiment using sonicated tumor lines. A, Subcutaneous tumor growth over time (left) and tumor weights (right) of 2838c3 WT tumors in either naïve mice (No immunization, n=8), mice injected four weeks prior with sonicated 2838c3 EV cells (dEV immunization, n=7), or mice injected four weeks prior with sonicated 2838c3 KO-A cells (dKO immunization, n=8). Statistical analysis done using two-way ANOVA. Data represent mean ± SEM.

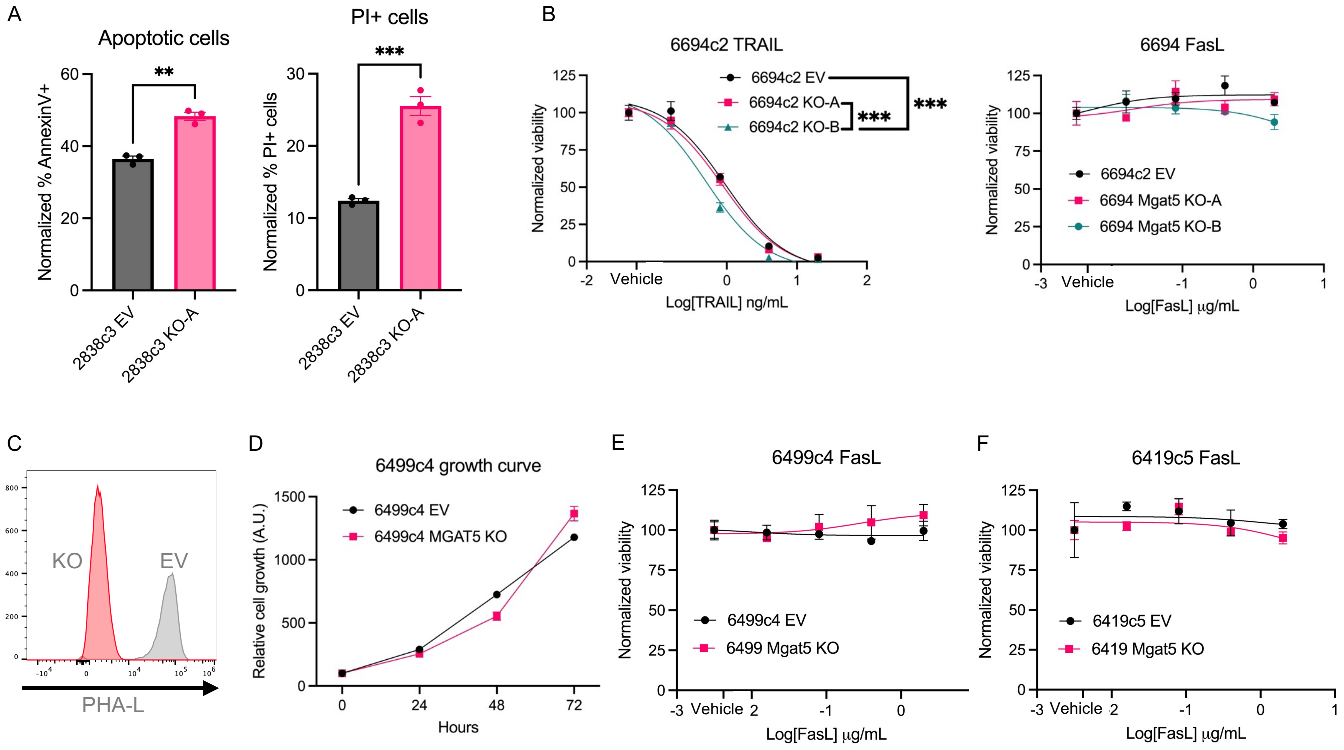

Figure S9: Full cell death panel of PDAC Mgat5 WT and KO lines. A, Cell death 24h after TNFA stimulation as measured by Annexin V / PI staining in 2838c3 EV and Mgat5 KO cell lines, normalized to cell alone controls. Statistics done using unpaired Student's *t* test. **B,** Cell death assays of 6694c2 EV and Mgat5 KO cells *in vitro* using varying concentrations of TRAIL and Fas ligand. Statistics from 0.8ng/mL TRAIL and 2µg/mL FasL. Analysis using two-way ANOVA for this and all further cell death figures at specified concentrations. **C,** Histogram of PHA-L binding to 6499c4 EV and Mgat5 KO cell lines. **E,** Relative growth of 6499c4 EV and Mgat5 KO cell lines *in vitro* over 72 hours (*n* = 3 replicates per data point). Statistical analysis using two-way ANOVA. **F,** Cell death assays of 6499c4 EV and Mgat5 KO cells *in vitro* using varying concentrations of Fas ligand. Statistics from 2µg/mL FasL. **G,** Cell death assays of 6419c5 EV and Mgat5 KO cells *in vitro* using varying concentrations of Fas ligand. Statistics from 2µg/mL FasL.

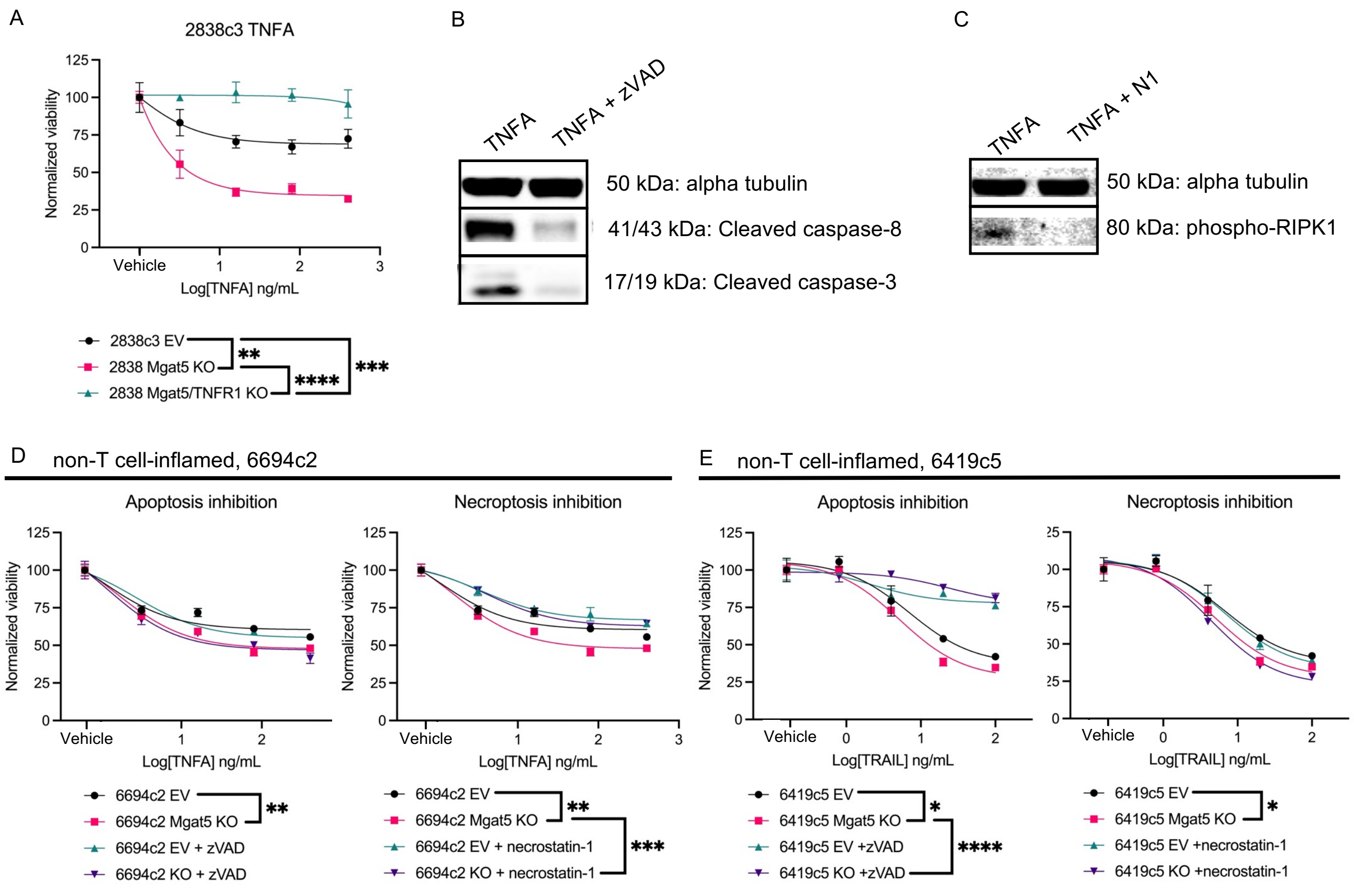

Figure S10: Full cell death rescue assays of PDAC Mgat5 WT and KO lines. A, Cell death assays of 2838c3 EV, Mgat5 KO, and TNFR1/Mgat5 DKO cells *in vitro* using varying concentrations of TNFA. Statistics from 80ng/mL TNFA using two-way ANOVA, for this and all following cell death assays at the specified concentrations. **B,** Western blot of 6499c4 EV cells stimulated with 40ng/mL TRAIL, IFNG, and CH for 24h with and without 10uM z-VAD-FMK pan-caspase inhibitor. **C,** Western blot of 2838c3 EV cells stimulated with 80ng/mL TNFA, IFNG, and CH for 24h, with and without 20uM necrostatin-1 pan-caspase inhibitor. **D,** Cell death assays of 6694c2 EV and Mgat5 KO cells *in vitro* using varying concentrations of TNFA, with and without addition of apoptosis inhibitor zVAD or necroptosis inhibitor necrostatin-1. Statistics from 80ng/mL TNFA. **E,** Cell death assays of 6419c5 EV and Mgat5 KO cells *in vitro* using varying concentrations of TRAIL, with and without addition of apoptosis inhibitor zVAD or necroptosis inhibitor necrostatin-1. Statistics from 80ng/mL TNFA.

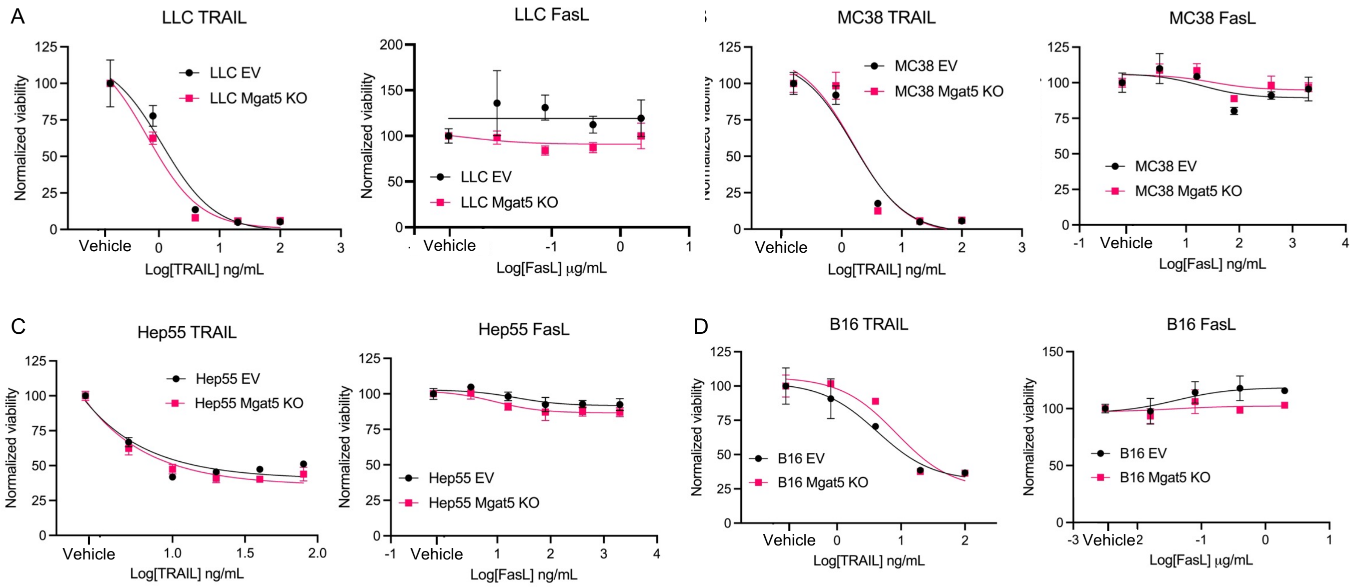

Figure S11: Full cell death panel of non-PDAC Mgat5 WT and KO cell lines. A, Cell death assays of LLC EV and Mgat5 KO cells *in vitro* using varying concentrations of TRAIL and Fas ligand. Statistics using two-way ANOVA for this and all following cell death assays. **B,** Cell death assays of MC38 EV and Mgat5 KO cells *in vitro* using varying concentrations of TRAIL and Fas ligand. **C,** Cell death assays of Hep55 EV and Mgat5 KO cells *in vitro* using varying concentrations of TRAIL and Fas ligand. **D,** Cell death assays of B16 EV and Mgat5 KO cells *in vitro* using varying concentrations of TRAIL and Fas ligand.

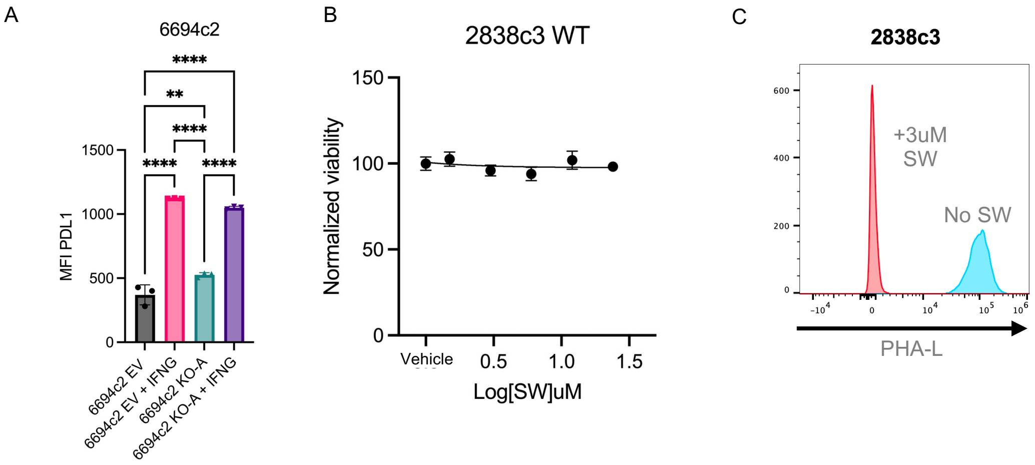

Figure S12: Therapeutic sensitivities of non-T cell-inflamed EV and Mgat5 KO tumors. **A,** MFI of PDL1 in 6694c2 EV and Mgat5 KO-A cells, with and without 100ng/mL IFNg pre-incubation for 24 hours. Statistical analysis done using one-way ANOVA. Error bars are SD. **B,** Normalized viability, as assessed by CellTiter Glo, of varying concentrations of swainsonine in 2838c3 WT after 72h of incubation. Error bars are SD. **C,** Flow cytometry for PHA-L of 2838c3 WT cells treated with and without 3μM swainsonine for 72 hours.
